# Heat acclimation defense against exertional heat stroke by improving the function of preoptic TRPV1 neurons

**DOI:** 10.7150/thno.101422

**Published:** 2025-01-01

**Authors:** Jing Li, Ziqing Zhou, You Wu, Jianshuai Zhao, Haokai Duan, Yuliang Peng, Xiaoke Wang, Zhongmin Fan, Lu Yin, Mengyun Li, Fuhong Liu, Yongheng Yang, Lixia Du, Jin Li, Haixing Zhong, Wugang Hou, Fanglin Zhang, Hongwei Ma, Xijing Zhang

**Affiliations:** 1 Department of Critical Care Medicine and Department of Anaesthesiology, Xijing Hospital, Fourth Military Medical University, Xi'an, Shaanxi, China, 710032.; 2 Institute of Biotechnology, Academy of Military Medical Sciences, Beijing, China, 100071.; 3 Department of Microbiology, School of Basic Medical Sciences, Fourth Military Medical University, Xi'an, Shaanxi, China, 710032.

**Keywords:** Exertional heat stroke, Heat acclimation, Medial preoptic area, TRPV1, Irisin, Exosome

## Abstract

**Rationale:** Record-breaking heatwaves caused by greenhouse effects lead to multiple hyperthermia disorders, the most serious of which is exertional heat stroke (EHS) with the mortality reaching 60 %. Repeat exercise with heat exposure, termed heat acclimation (HA), protects against EHS by fine-tuning feedback control of body temperature (Tb), the mechanism of which is opaque. This study aimed to explore the molecular and neural circuit mechanisms of the HA training against EHS.

**Methods:** Male C57BL/6 mice (6-8 weeks) and male TRPV1-Cre mice (6-8 weeks) were used in our experiments. The EHS model with or without HA training were established for this study. RNA sequencing, qPCR, immunoblot, immunofluorescent assays, calcium imaging, optogenetic/ chemical genetic intervention, virus tracing, patch clamp, and other methods were employed to investigate the molecular mechanism and neural circuit by which HA training improves the function of the medial preoptic area (mPOA) neurons. Furthermore, a novel exosome-based strategy targeting the central nervous system to deliver irisin, a protective peptide generated by HA, was established to protect against EHS.

**Results:** HA-related neurons in the mPOA expressing transient receptor potential vanilloid-1 (TRPV1) were identified as a population whose activation reduces Tb; inversely, dysfunction of these neurons contributes to hyperthermia and EHS. mPOA^TRPV1^ neurons facilitate vasodilation and reduce adipose tissue thermogenesis, which is associated with their inhibitory projection to the raphe pallidus nucleus (RPa) and dorsal medial hypothalamus (DMH) neurons, respectively. Furthermore, HA improves the function of preoptic heat-sensitive neurons by enhancing TRPV1 expression, and *Trpv1* ablation reverses the HA-induced heat tolerance. A central nervous system-targeted exosome strategy to deliver irisin, a protective peptide generated by HA, can promote preoptic TRPV1 expression and exert similar protective effects against EHS.

**Conclusions:** Preoptic TRPV1 neurons could be enhanced by HA, actively contributing to heat defense through the mPOA"DMH/RPa circuit during EHS, which results in the suppression of adipose tissue thermogenesis and facilitation of vasodilatation. A delivery strategy of exosomes engineered with RVG-Lamp2b-Irisin significantly improves the function of mPOA^TRPV1^ neurons, providing a promising preventive strategy for EHS in the future.

## Introduction

Heat stroke (HS), including classic heat stroke (CHS) and exertional heat stroke (EHS), is a life-threatening systemic disease characterized by extreme hyperthermia (core temperatures usually exceeding 40.5 °C), rapid onset by central nervous system (CNS) abnormalities and subsequent multiple organ dysfunction 1. Compared with CHS, EHS maintains severer disease progression and higher mortality, which is caused by complex factors including high-temperature and high-humidity environments with high-intensity physical activity, and is prevalent in athletes, officers, and soldiers 2,3. High-intensity physical activity promotes muscular heat accumulation and high-humidity environments prevent cooling efficiency of sweating, thus boosting body temperature (Tb). And hyperthermia directly contributes to EHS pathogenies by inducing systemic inflammatory responses 4, multiple programmed cell death patterns 3, oxidative stress 5 and coagulation disorders 6, resulting in irreversible tissue injury and organ dysfunction 7. Consistent deterioration of global warming leads to increasing incidence rate of EHS patients annually, the fatality rate of which reaches 60 % 2. To date, no specific and effective intervenes but reducing Tb are available for EHS in clinical practice 8. Although it has been confirmed that heat acclimation (HA) could maintain Tb homeostasis and prevent EHS during heat defense, the specific mechanism of how HA modulates thermoregulatory circuits still remains obscure.

Heat defense is achieved by elaborate coordination between the central nucleus and peripheral effector tissues 9,10. The medial preoptic area (mPOA) serves as a central integratory hub to regulate Tb in cold or warm ambient environments, as well as influencing sleeping, parenting, reproductive and other reward behavior 11-13. Thermosensitive neurons in various POA subregions control homeothermy by driving heat dissipation, generation or conservation. Dorsomedial hypothalamus (DMH) is a pivotal nucleus in the output pathway of mPOA neurons, the projection of which can be the disinhibition of an inhibitory POA to DMH, or the enhancement of an excitatory mPOA to DMH 9,14,15. The subsequent thermoregulatory effects of DMH on peripheral effector tissues are the modulation of adipose tissue thermogenesis, or the manipulation of vascular dilatation or constriction through cardiovascular sympathetic outflow 16-18. Heat defense activities, including induction of vasodilation, reduction of energy expenditure or interscapular brown adipose tissue (iBAT) thermogenesis, and suppression of muscle shivering activity, are highly involved with the mPOA"DMH, mPOA"raphe pallidus nucleus (RPa) or DMH" RPa circuits 19. Of note, warm-sensitive preoptic neurons exhibit high heterogeneity, expressing specific or overlapped markers such as *Bdnf, CCK, ERα, galanin, Pgds2, Qrfp, Nos1, Vglut2/Pacap/leptin* receptor, *Vgat, Trpm2* and *Trpc4* 20-27. Recent researches indicate that HA can enhance the acquired thermal adaptation ability by promoting the proliferation and differentiation of nascent neurons in the mPOA, while the neuronal identity and circuitry of HA-improved mPOA neurons are poorly understood 28. Furthermore, it has been reported that several muscle-derived factors released into the circulation upon physical exercise, including irisin, muslin, brain-derived neurotrophic factor (BDNF) and cathepsin B (CTSB), exert neuroprotective effects 29-34. Previous studies identify irisin as a cleaved myokine from fibronectin type III domain containing 5 (FNDC5) in muscle under the control of peroxisome proliferator-activated receptor-γ coactivator 1α (PGC-1α), which shifts the adipose metabolism toward a thermogenic profile during exercise 35,36. Intriguingly, recent researches demonstrate that irisin in mice brains could be induced via PGC-1α, which rescues synaptic plasticity and memory defects in Alzheimer's disease mice model by stimulating the expression of BDNF in the hippocampus 29,37. Additionally, post treatment of irisin after intracerebral hemorrhage also ameliorates neuroinflammation and restrains neuronal apoptosis through the integrin αVβ5/AMPK pathway 38. Yet, it is not clear whether irisin participates in the thermoregulatory process during heat defense.

Transient receptor potential vanilloid-1 (TRPV1), an important member of the transient receiver potential (TRP) family, is a kind of non-selective channel protein responsible for decoding physical and chemical stimuli 39. TRPV1^+^ neurons mainly distribute in peripheral sites such as the skin or dorsal root ganglion, which are activated by the stimulation of capsaicin, acid or heat (more than 43 °C), mediating the signals of pain, inflammation or Tb 40,41. Nevertheless, increasing evidence suggests TRPV1 expression in specific brain regions and its potential role in psychiatric disorders 42. Here, we elucidate that preoptic TRPV1 neurons, the function of which could be enhanced by HA via irisin, actively contribute to heat defense during EHS. mPOA^TRPV1^ neurons form inhibitory circuits with downstream DMH and RPa neurons, restraining the iBAT thermogenesis and facilitating mice tail vasodilation, respectively. A delivery strategy of exosomes engineered with RVG-Lamp2b-Irisin significantly improves the function of preoptic TRPV1 neurons and prevents EHS.

## Results

### HA training enhances heat tolerance and improves neurological prognosis of EHS

**Figure 1:**
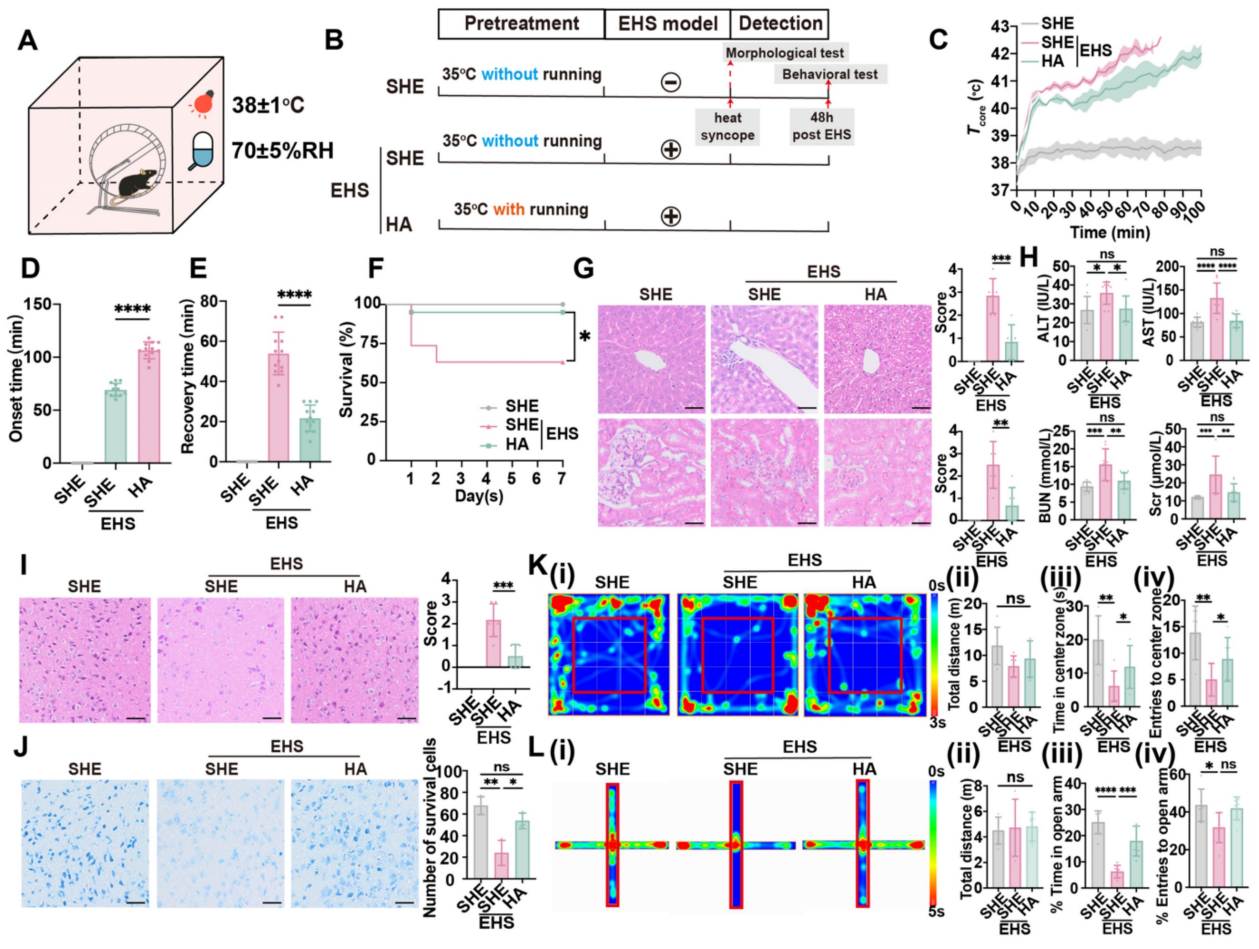
** HA improves heat tolerance and EHS outcome.** (A) Schematics of EHS model. (B) Training timeline for the separate heat exposure (SHE) group, exertional heat stroke (EHS) group, and heat acclimation (HA) group. (C) The changes of *T*core during the modeling process. (D-E) The onset time of heat-syncope (D) and recovery time post EHS (E) (n = 12). (F) 7-day mortality rate in each group of mice (n = 20). (G) HE staining of the liver and kidney and the histological damage score for HE staining images evaluation (n = 6). (H) Results of the serum concentration of alanine aminotransferase (ALT) and aspartate aminotransferase (AST), blood urea nitrogen (BUN) and serum creatinine (Scr) (n = 10). (I) HE staining of the brain and the histological damage score for HE staining images evaluation (n = 6). Scale bar, 100 μm. (J) Nissl staining of the brain and the number of survival cells for Nissl staining images evaluation (n = 3). Scale bar, 100 μm. (K) Typical tracking chart of the open field test (OFT) (i), statistics of total distance travelled (ii), time in the center zone (iii), and the number of entries to the center zone (iv) in the OFT at 48 h of recovery (n = 6). (L) Typical tracking chart of the elevated plus maze (EPM) (i), statistics of total distance travelled (ii), time in the open arm (iii), and the number of entries to the open arm (iv) in the EPM at 48 h of recovery (n = 6). All data are shown as mean ± SD and were analyzed by one-way ANOVA, or two-way ANOVA. *p < 0.05, **p < 0.01, ***p < 0.001, ****p < 0.0001, ns, not significant.

An EHS murine model with or without HA training was established according to previous study 43,44, and separate heat exposure (SHE) was set as the control against HA before EHS challenge (Figure 1A and 1B). Although both SHE and HA groups experienced a rapid core temperature elevation from 37 to 40 °C within 10 min, HA training prevented hyperthermia (> 41 °C) from 10 to 60 min when mice were exposed to the EHS environment (Figure 1C). Heat-syncope onset time and recovery time were measured to evaluate the heat tolerance ability post EHS (Figure 1D and 1E). HA training group exhibited remarkably delayed heat-syncope onset time than the SHE group (106.5 ± 7.914 min vs 69.17 ± 6.118 min with control, *p* < 0.0001, Figure 1D). The recovery time of EHS mice was also significantly shortened in the HA training group (21.67 ± 6.569 min vs 53.92 ± 10.62 min with control, *p* < 0.0001, Figure 1E). To further investigate the role of HA training on EHS outcome, the fatality rate and other indicators for multiple organ dysfunction were detected (Figure 1F-1K). The EHS mice maintained a survival rate of 63.16 %, which was increased to 95.00 % by HA training (Figure 1F). Severe immunopathological injuries were detected by HE staining for liver and kidney in the EHS model, which would be alleviated by HA pretreatment (Figure 1G). The clinical parameters for liver and kidney injury, including serum concentration of alanine aminotransferase (ALT) and aspartate aminotransferase (AST), blood urea nitrogen (BUN) and serum creatinine (Scr), respectively, significantly increased in the EHS model, while most of them maintained in a relatively lower level in the HA group (Figure 1H). Moreover, severe morphological injuries were detected by HE staining and Nissl staining for the brain in the EHS model, which would be alleviated by HA pretreatment (Figure 1I and Figure 1J). These data indicated that HA could enhance heat resistance and improve EHS prognosis in mice.

It has been reported that there existed cerebral dysfunction post EHS 1,45, and, here, different behavioral models were applied to evaluate the neurological prognosis. Firstly, contextual fear conditioning and Barnes maze tests were exploited to measure the cognitive function (Figure S1A-S1E). Latency to reach the target region in the Barnes maze test of the EHS group showed no alteration compared with the control group from 3 to 6 days post spatial learning (Figure S1E); additionally, the total distance and time in the target zone in the Barnes maze test also remained unchanged of different groups (Figure S1B-S1D), which suggested that the spatial learning ability was not affected by EHS. The freezing time of EHS mice did not change compared with the control group (Figure S1F and S1G), indicating that no memory deficits occurred post EHS. Secondly, the rotarod test was used to detect cerebellar dysfunction. The endurance time of rotarod test did not change post EHS (Figure S1H and S1I), showing that the motor coordination ability was not affected by EHS. Furthermore, open field maze (OFM) and elevated plus-maze (EPM) tests were applied to assess anxiety-like behavior and exploratory activity (Figure 1K-1L). The total distance travelled in OFM remained unchanged in different groups, while both time and number of entries to the center zone significantly collapsed post EHS (Figure 1K). Consistently, both the time in and entries to the open arm of EHS mice decreased compared with the SHE group (Figure 1L). These data suggested that EHS mice showed anxiety-like behavior and reduced exploratory activity. HA training could efficiently improve the parameters of OFM and EPM tests (Figure 1K-1L), indicating that HA might ameliorate neurological injury by EHS.

### Preoptic TRPV1 neurons response to EHS condition that could be regulated by HA training

**Figure 2:**
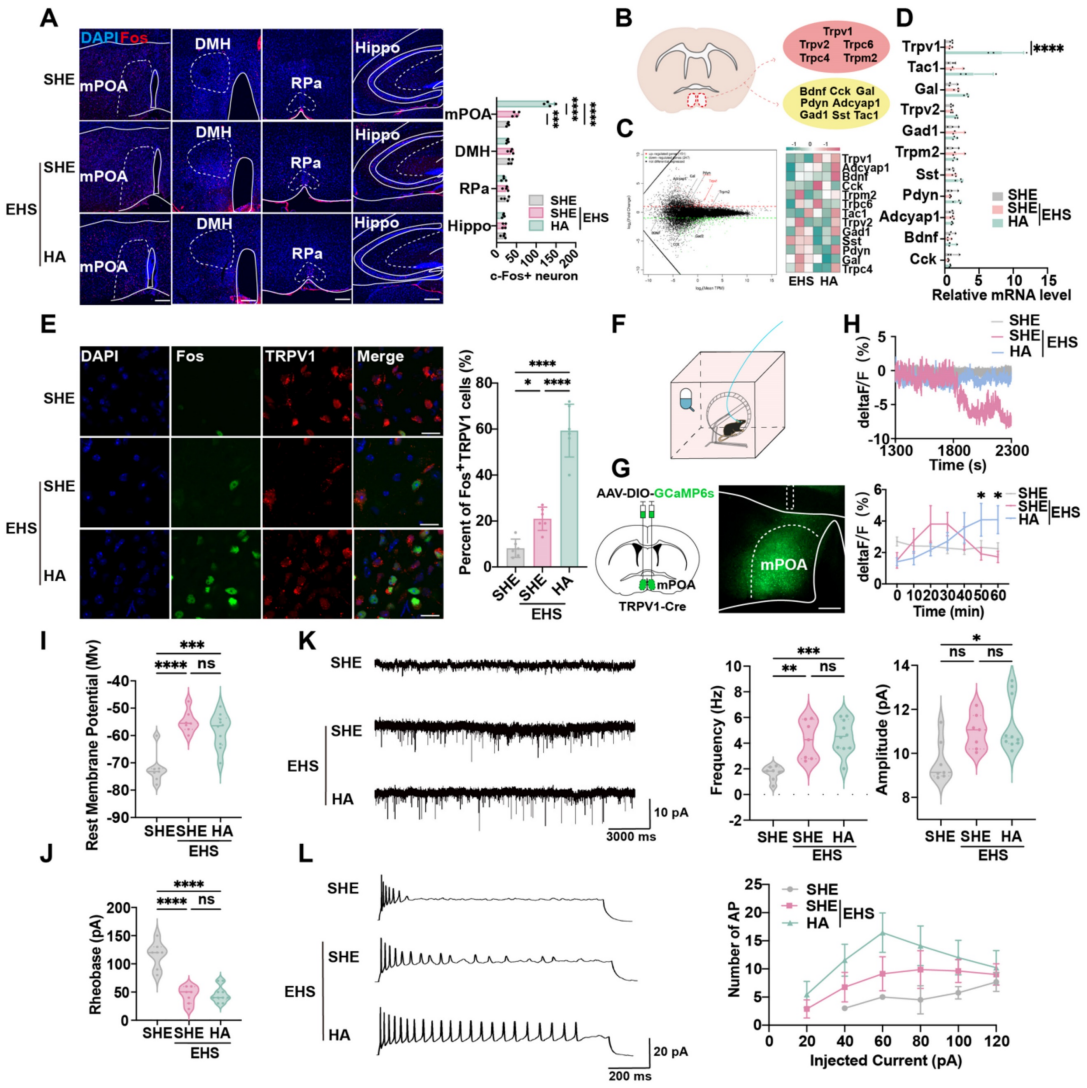
** TRPV1 in the mPOA is required for the protective effect of HA.** (A) Representative confocal images and analysis of Fos in mPOA, DMH, RPa, hippocampus in SHE, EHS, and HA mice. DAPI, blue; Fos, red. Scale bar, 100 μm (n = 4). (B) Scheme of sampling region in RNA sequence experiment and candidate gene markers. (C) Gene expression difference MA diagram and heatmap. The horizontal axis represents the mean log2 (TPM) of two sets of samples, i.e. (log2 (A)+log2 (B))/2, while the vertical axis represents log2 (Foldchange), i.e. log2(B/A) value. Each point in the graph represents a gene, with red indicating upregulated genes, green indicating downregulated genes, and black indicating non-differentially expressed genes. TRPV1 as indicated by the red arrow. (D) The result of qPCR of genetic markers including *TRPV1, Tac1, Gal, Trpv2, Gad1, Trpm2, Sst, Pdyn, Adcyap1, Bdnf*, and *Cck* (n = 3). (E) Representative confocal images and average percentages of Fos in TRPV1- expressed neurons. DAPI, blue; Fos, green; TRPV1, red. Scale bar, 20 μm. White arrows indicate cells with co-localization of Fos and TRPV1 (n = 6). (F) Scheme of AAV-mediated GCaMP6s expression (green) in TRPV1-Cre mice. (G) Representative image of AAV-mediated GCaMP6s expression (green) in TRPV1- Cre mice. White lines indicate the boundary of the brain, mPOA, or the implanted fiber. Scale bar, 100 μm. (H) Representative mPOA^TRPV1^ GCaMP6s fluorescence signal during modeling and the quantification of mPOA^TRPV1^ GCaMP6s fluorescence every ten minutes (n = 4). (I-J) Analysis of rest membrane potential and rheobase of mPOA^TRPV1^ neuron in the SHE group (n = 7), the SHE+EHS group (n = 7) and the HA+EHS group (n = 10). (K) Schematic illustration and the analysis of EPSP in the SHE group (n = 7), the SHE+EHS group (n = 7) and the HA+EHS group (n = 10). (L) Schematic illustration and the analysis of AP in the SHE group (n = 7), the SHE+EHS group (n = 7) and the HA+EHS group (n = 10). All data are shown as mean ± SD and were analyzed by one-way ANOVA, or two-way ANOVA. *p < 0.05, **p < 0.01, ***p < 0.001, ****p < 0.0001, ns, not significant.

Whole-brain staining and scanning assays were performed to investigate the central nervous mechanism by which HA training reinforces heat resistance. The thermoregulatory region mPOA, but not DMH, RPa, or memory-related hippocampus (Hippo), showed a significant increase in Fos^+^ cells following EHS at 70 min post EHS treatment, which also was strengthened by HA training (Figure 2A). This indicated that certain heat-sensitive neurons might be activated by HA. RNA sequencing (RNA-seq) assays were used to identify the gene expression characteristics of neurons in mPOA reshaped by HA (Figure 2B). Previous research suggested that thermosensitive neurons could be marked by *Pdyn, Bdnf, Cck, Adcyap1, Tac1, Sst, Gal* and *Gad1*, as well as the thermosensitive channel proteins such as *Trpv1, Trpv2*, and *Trpm2* 27,27,46-51. Our gene ontology (GO) analysis demonstrated that in the HA group, the upregulated genes were primarily involved in ion transport (Figure S2A). Kyoto Encyclopedia of Genes and Genomes (KEGG) analysis also revealed that the enriched pathways in the HA group mainly participated in regulating TRP channels (Figure S2B). The euKaryotic Ortholog Groups (KOG) analysis showed that HA training altered the expression of genes involved in inorganic ion transport and metabolism (Figure S2C). Intriguingly, among the foresaid genes, we found that HA training could significantly promote the mRNA expression of *Trpv1*, an important member of TRP family, but not other markers compare with the SHE group (Figure 2C), the alteration of which was also confirmed by the real-time quantitative PCR (qPCR) assays (Figure 2D).

To evaluate whether mPOA^TRPV1^ neurons were activated by hyperthermia, the c-Fos staining (Figure 2E) and calcium signal detection (Figure 2F-2H) assays were performed. The results showed that TRPV1^+^ neurons expressed c-Fos at 70 min post EHS treatment, and the percentage of Fos^+^ in mPOA^TRPV1^ neurons was higher in HA training mice compared with the EHS group (133.5 ± 12.61 min vs 48.0 ± 7.53 min with control, *p* < 0.0001, Figure 2E), indicating that the activation of mPOA^TRPV1^ neurons might help protect against EHS. The GCaMP6 calcium indicators for neuronal imaging assays suggested that the electrical activity of the SHE group collapsed since 1800 s post EHS treatment, while HA training mice showed sustained activation of mPOA^TRPV1^ neurons from 1300 to 2300 s post EHS exposure (Figure 2F-2H). These data indicated that HA could improve the function of mPOA^TRPV1^ neurons under EHS conditions.

To detect the mechanism of how HA training influenced neuron function, the whole-cell patch clamp technique was used to investigate the electrophysiological characteristics of mPOA^TRPV1^ neurons. The AAV2/9-DIO-mCherry virus was injected into the mPOA of TRPV1-Cre mice, and mCherry-positive mPOA^TRPV1^ neurons were targeted for patch clamp assays. EHS exposure increased the rest potential, but decreased the rheobase of mPOA^TRPV1^ neurons compared with the control group (Figure 2I and 2J). Moreover, both the frequency and amplitude of excitatory post-synaptic currents (EPSCs) were enhanced post EHS modeling (Figure 2K). These results showed that hyperthermia enhanced the excitability of preoptic TRPV1 neurons, however, this was not more strengthened by HA training. To note, the action potential (AP) number of mPOA^TRPV1^ neurons increased in the EHS group compared with SHE, which was further upregulated in the HA-pretreated EHS group compared with the SHE-pretreated EHS (Figure 2L). Taken together, HA training improved the function of preoptic TRPV1 neurons not by directly altering their membrane potential or EPSCs, but by enhancing their sensitivity for stimulation and ability to generate action potential.

### mPOA^TRPV1^ " DMH neurons are mixed glutamatergic and GABAergic populations

**Figure 3:**
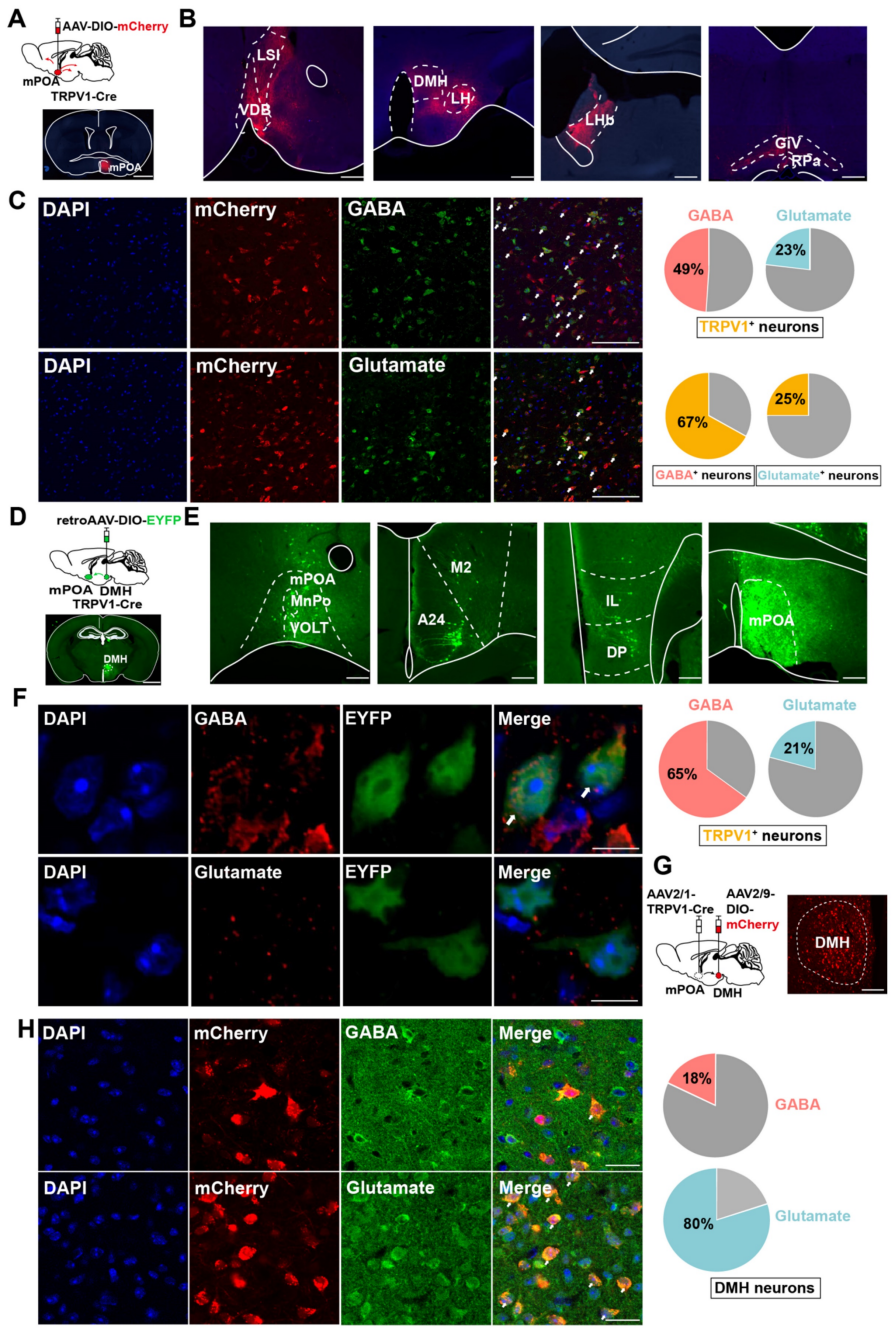
** mPOA^TRPV1^** → **DMH neurons are mixed glutamatergic and GABAergic populations.** (A) Scheme and representative coronal images of the injection of AAV-DIO-mCherry into mPOA of TRPV1-Cre mice. Scale bar, 500 µm. (B) Representative images of output from TRPV1 neurons in mPOA. Scale bar, 100 µm. (C) Overlap between TRPV1-expressed neurons labeled by mCherry (red) and GABAergic or glutamatergic neurons (green) in mPOA. Scale bar, 50 µm. White arrows indicate cells with co-localization of TRPV1 and GABA /Glutamate (n = 6). (D) Schematic and representative coronal images of the injection of Cre-independent AAVretro into the DMH. Scale bar, 500 µm. (E) Representative images of TRPV1-expressing neurons outputting to DMH. Scale bar, 100 µm. (F) Overlap between TRPV1-expressed neurons labeled by EYFP (green) and GABAergic or glutamatergic neurons (red) in mPOA. Scale bar, 4 μm. White arrows indicate cells with co-localization of TRPV1 and GABA /Glutamate (n = 6). (G) Schematic and representative coronal images of the injection of AAV1-TRPV1-Cre into the mPOA and AAV-DIO-mCherry into the DMH of wide-type mice. Scale bar, 50 µm. (H) Overlap between TRPV1-projecting neurons labeled by mCherry (red) and GABAergic or glutamatergic neurons (green) in the DMH. Scale bar, 20 µm. White arrows indicate cells with co-localization of mCherry and Glutamate/GABA (n = 3).

As preoptic TRPV1 neurons were activated by the EHS condition (Figure 2), we investigated the integration of mPOA^TRPV1^ in the thermoregulation circuit by tracking the mCherry-labeled preoptic TRPV1 axonal fibers (Figure 3A). mPOA^TRPV1^ neurons were labeled by injections of the AAV2/9-DIO-mCherry into TRPV1-Cre mice (Figure 3A). Projections to 7 brain areas were identified (Figure 3B) that included areas related to thermoregulation (especially DMH and RPa), as well as stress, emotion, appetite and sleep, thus representing possible nodes at which mPOA^TRPV1^ neurons may exert their function during EHS model. Next, we assessed the neurotransmitters used by mPOA^TRPV1^ neurons. Approximately 49 % (± 7.43 %) of TRPV1 neurons were positive for GABA, with nearly 67 % (± 7.69 %) of GABAergic neurons expressing TRPV1 (Figure 3C). In comparison, 23 % (± 8.32 %) TRPV1 neurons were positive for glutamate, and 25 % (± 9.92 %) glutamatergic neurons expressed TRPV1 (Figure 3C).

To test whether mPOA^TRPV1^ neurons form direct connections with the effector nucleus for thermoregulation, we used retroAAV-DIO-EYFP tracing from DMH and measured retrograde signals of mPOA^TRPV1^ neurons in TRPV1-Cre mice (Figure 3D). Consistent with previous tracing results, the retrograde assays showed that EYFP expressed in mPOA, confirming the projection from mPOA^TRPV1^ to DMH (Figure 3E). Additionally, we also found that mPOA^TRPV1^ neurons projecting to DMH overlapped with more GABA (65 ± 7.01 %) than glutamate (21 ± 6.68 %) (Figure 3F). Then, the neuron types of DMH receiving projection from mPOA^TRPV1^ were assessed. The AAV2/1-TRPV1-Cre and AAV2/9-DIO-mCherry viruses were injected into the mPOA and DMH, respectively (Figure 3G), and the GABA and glutamate were stained in DMH (Figure 3H). We found that the DMH neurons receiving projection from the mPOA^TRPV1^ neurons exhibited a higher colocalization rate with glutamate (80 ± 7.90 %) but lower rate with GABA (18 ± 8.11 %) (Figure 3H). Thus, mPOA^TRPV1^"DMH neurons are mixed glutamatergic and GABAergic populations. And according to the colocalization rate, mPOA^TRPV1^ neurons mainly provide inhibitive projections to DMH glutamatergic neurons, which means that the activation of preoptic TRPV1 neurons might restrain the thermogenesis effects of DMH.

### Optogenetic or chemogenetic activation of mPOA^TRPV1^ neurons decreases Tb and physical activity

**Figure 4:**
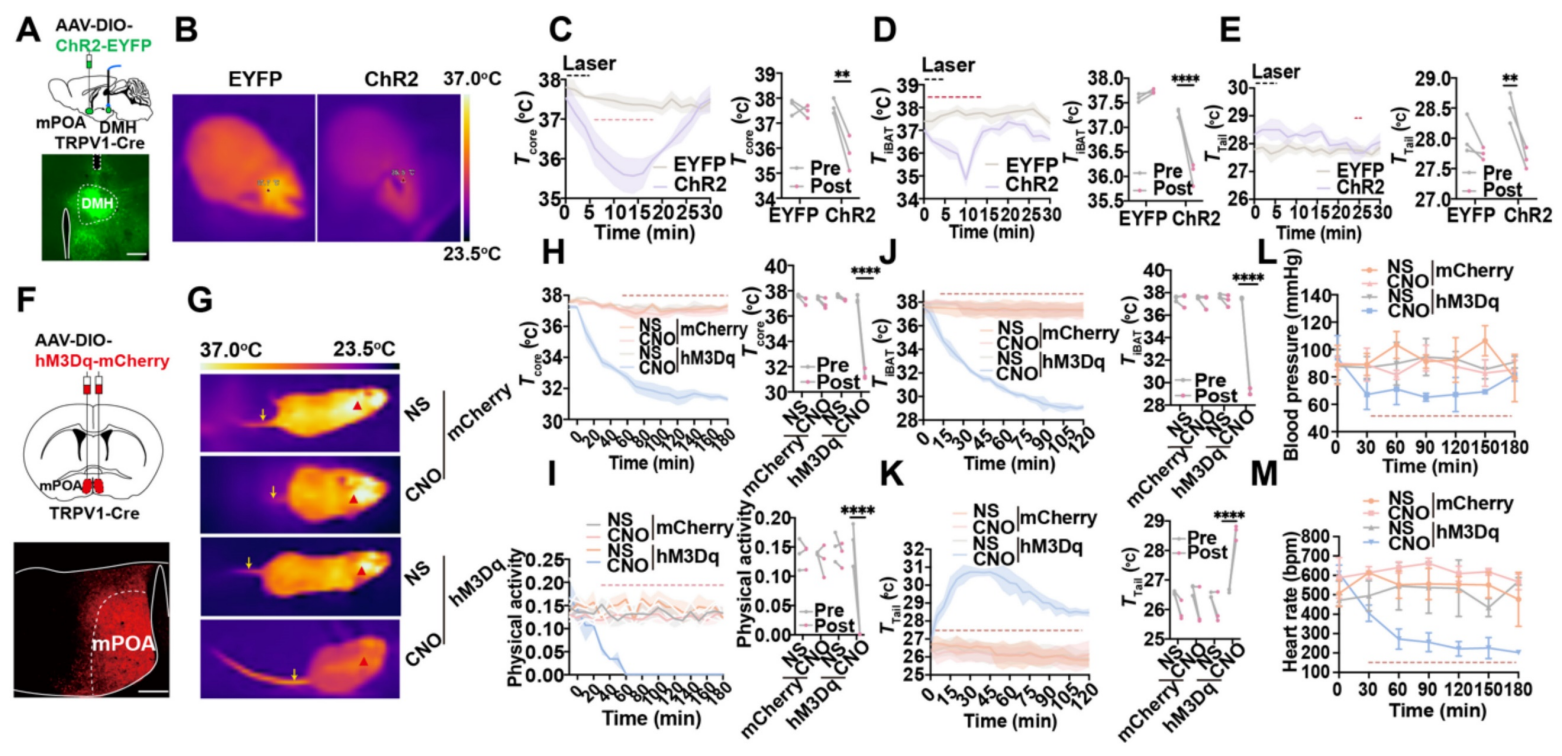
** Activation of mPOA**^TRPV1^** neurons leads to thermogenesis.** (A) Scheme and representative coronal images of the injection of AAV-DIO-ChR2- EYFP virus into the mPOA and placement of optic fiber. (B) Representative images of infrared thermal images after laser stimulation. (C) Average traces for changes in *T*core. Quantitation in right panels (intervals: off, -10 to 0 min; on, 6 to 16 min, n = 3). (D) Average traces for changes in *T*iBAT. Quantitation in right panels (intervals: off, -10 to 0 min; on, 4 to 16 min, n = 3). (E) Average traces for changes in *T*_tail_. Quantitation in right panels (intervals: off, -2 to 0 min; on, 24 to 26 min, n = 3). (F) Schematic and representative coronal images of the injection of AAV-DIO-hM3D(Gq)-mCherry into mPOA. Scale bar, 200 µm. (G) Representative infrared thermal images with or without chemogenetic activation of mPOA^TRPV1^ neurons. (H-I) Average traces for *T*_core_ (H) and physical activity (I) change with chemogenetic activation. Quantitation in right panels (intervals: off, -30 to 0 min; on, 150 to 180 min, n = 3). (J-K) Average traces for changes in *T*_iBAT_ (J) and *T*tail (K) with chemogenetic activation. Quantitation in right panels (intervals: off, -30 to 0 min; on, 90 to 120 min, n = 3). (L-M) Average traces for changes in blood pressure (L) and heart rate (M) with chemogenetic activation (n = 3). All data are shown as mean ± SD and were analyzed by one-way ANOVA, two-way ANOVA, or Paired t tests. *p < 0.05, **p < 0.01, ***p < 0.001, ****p < 0.0001, ns, not significant.

Optogenetic activation of mPOA^TRPV1^ neurons allowed us to study the rapid physiological responses without handling the mice, which could be used to investigate the role of mPOA^TRPV1^ neurons on basal body temperature regulation. AAV-DIO-ChR2-EYFP or control (AAV-DIO-EYFP) was injected into the mPOA of TRPV1-Cre mice, and optical fibers were embedded in the DMH area (Figure 4A). *In vivo*, awake mice in their home cages were studied during three cycles consisting of 5 min of optostimulation followed by 25 min with the laser off. We monitored the core temperature (*T*_core_, measured intraperitoneally with DSI) together with the surface temperature of iBAT (*T*_iBAT_) and tail skin (*T*_tail_) after exposing mice at room temperature (Figure 4B). Optogenetic activation of the projection terminals of POA^TRPV1^ neurons to DMH could trigger a rapid, robust decrease in *T*_core_ (1.867 ± 0.394 °C vs 0.2 ± 0.069 °C with control, *p* = 0.0015; Figure 4C) and *T*_iBAT_ (1.239 ± 0.142 °C vs 0.133 ± 0.039 °C with control, *p* < 0.0001; Figure 4D), with an increase in *T*_tail_ (0.833 ± 0.074 °C vs 0.283 ± 0.221 °C with control, *p* = 0.0041; Figure 4E). This indicated *T*_iBAT_ process was suppressed, and the vasodilation power was increased when the mPOA^TRPV1^ " DMH circuit was activated. There was no attenuation of or systematic difference in *T*_core_ as a function of cycle number, so the three cycles were averaged in all further experiments. No effect was observed in EYFP^+^ control mice (Figure 4B-4E).

Next, chemogenetic assays were applied to selectively activate mPOA^TRPV1^ neurons and assess their roles in Tb regulation. Viral expression for the excitatory designer receptor exclusively activated by designer drug (DREADD) hM3Dq was confirmed (Figure 4F). Activation of mPOA^TRPV1^ neurons with CNO (1 mg/kg) decreases the *T*_core_ by 5.933 ± 0.114 °C (vs 0.344 ± 0.059 °C with control, *p* < 0.0001; Figure 4H), the physical activity (0.155 ± 0.037 vs 0.012 ± 0.003 with control, *p* < 0.0001; Figure 4I), and the *T*_iBAT_ by 8.352 ± 0.237 °C (vs 0.377 ± 0.349 °C with control, *p* < 0.0001; Figure 4G and 4J), with increasing the *T*_tail_ by 1.976 ± 0.184 °C (vs -0.471 ± 0.426 °C with control, *p* < 0.0001; Figure 4G and 4K). To note, chemogenetic activation of mPOA^TRPV1^ neurons also downregulated the blood pressure and heart rate (Figure 4L-4M). These results demonstrated that optogenetic or chemogenetic activation of mPOA^TRPV1^ neurons could efficiently reduce mice core temperature by suppressing iBAT thermogenesis and facilitating vasodilatation without affecting the physical activity.

### Chemogenetic activation of mPOA^TRPV1^ neurons exhibits preventive effects against EHS

**Figure 5:**
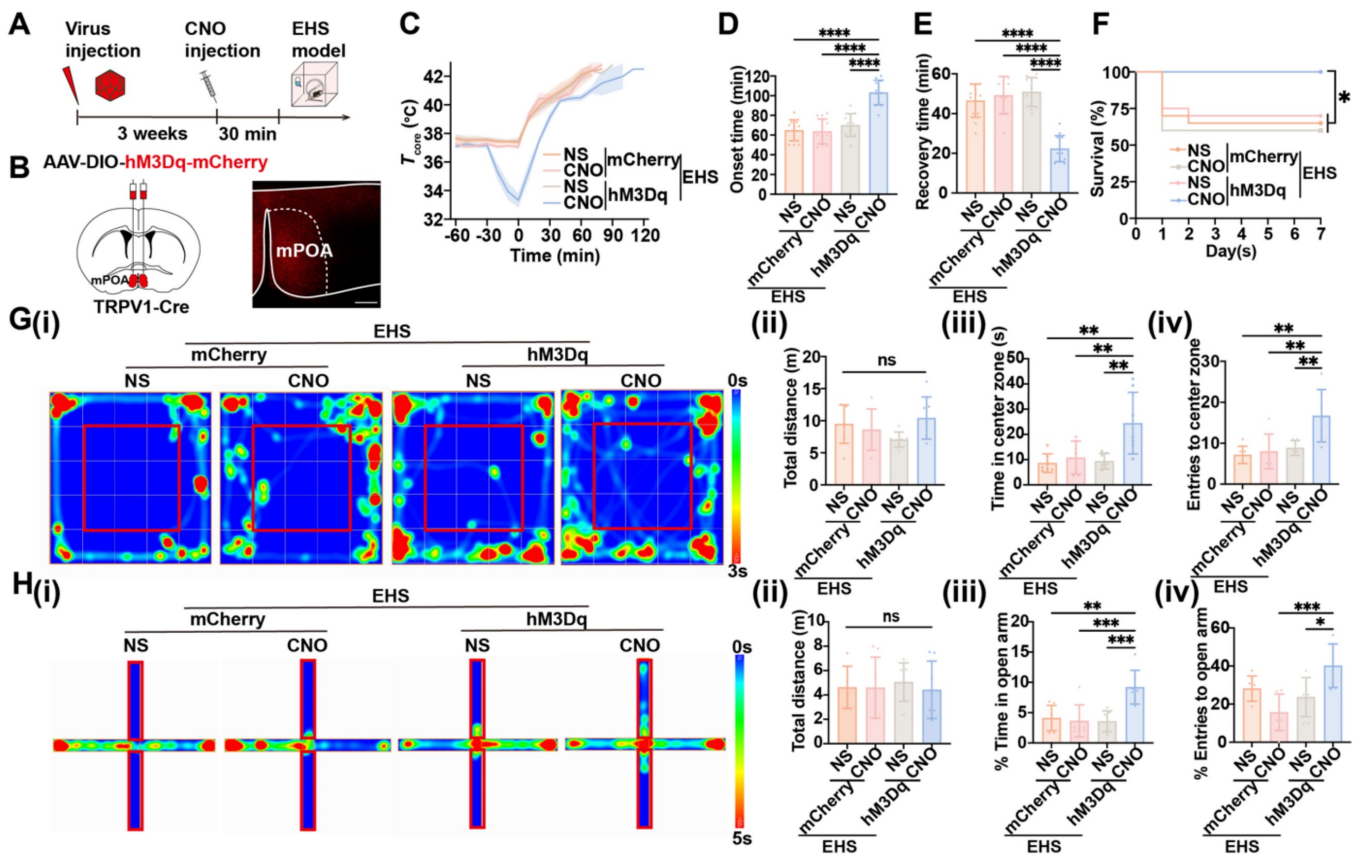
** Activation of mPOA^TRPV1^ neurons exhibits preventive effects against EHS.** (A) Scheme of the injection of virus and CNO. (B) Scheme and representative coronal images of the injection of AAV-DIO- hM3D(Gq)-mCherry into mPOA. Scale bar, 100 µm. (C) The changes of *T*core during the modeling process. (D-E) The onset time of heat-syncope (D) and recovery time post EHS (E) (n = 12). (F) 7-day mortality rate in each group of mice (n = 20). (G) Typical tracking chart of the OFT (i), statistics of total distance travelled (ii), time spent in the center zone (iii), and the number of entries to the center zone (iv) in the OFT at 48 h of recovery (n = 7). (H) Typical tracking chart of the EPM (i), statistics of total distance travelled (ii), time spent in the open arm (iii), and the number of entries to the open arm (iv) in the EPM at 48 h of recovery (n = 7). All data are shown as mean ± SD and were analyzed by one-way ANOVA, or two-way ANOVA. *p < 0.05, **p < 0.01, ***p < 0.001, ****p < 0.0001, ns, not significant.

Next, chemogenetics were applied to selectively activate mPOA^TRPV1^ neurons and assess their roles in heat defense (Figure 5A). Viral expression for the DREADD hM3Dq was confirmed (Figure 5B). Under the EHS circumstances, activation of mPOA^TRPV1^ neurons with CNO (1 mg/kg) before EHS model decelerated the *T*_core_ elevate (Figure 5C), increased the onset time of heat-syncope (103.30 ± 12.50 min vs 63.75 ± 12.61 min, 70.17 ± 11.65 min, and 64.83 ± 10.57 min with control, *p* < 0.0001, Figure 5D), and shortened the recovery time (22.33 ± 6.54 min vs 50.08 ± 10.66 min, 51.75 ± 8.63 min, and 46.50 ± 8.33 min with control, *p* < 0.0001, Figure 5E). The mortality rate also deteriorated in the TRPV1 activation group (Figure 5F). The total distance travelled in OFM was undifferentiated among all groups, while both time and number of entries to the center zone significantly collapsed in the TRPV1 activation group (Figure 5G). Consistently, both the time in and entries to the open arm of the TRPV1 activation mice decreased compared with the scramble group in the EPM tests (Figure 5H). These results indicated that chemogenetic activation of mPOA^TRPV1^ neurons exhibited preventive effects against EHS.

### TRPV1 facilitates the activation of warm-sensitive neurons in mPOA and is critical for HA-triggered heat resistance

**Figure 6:**
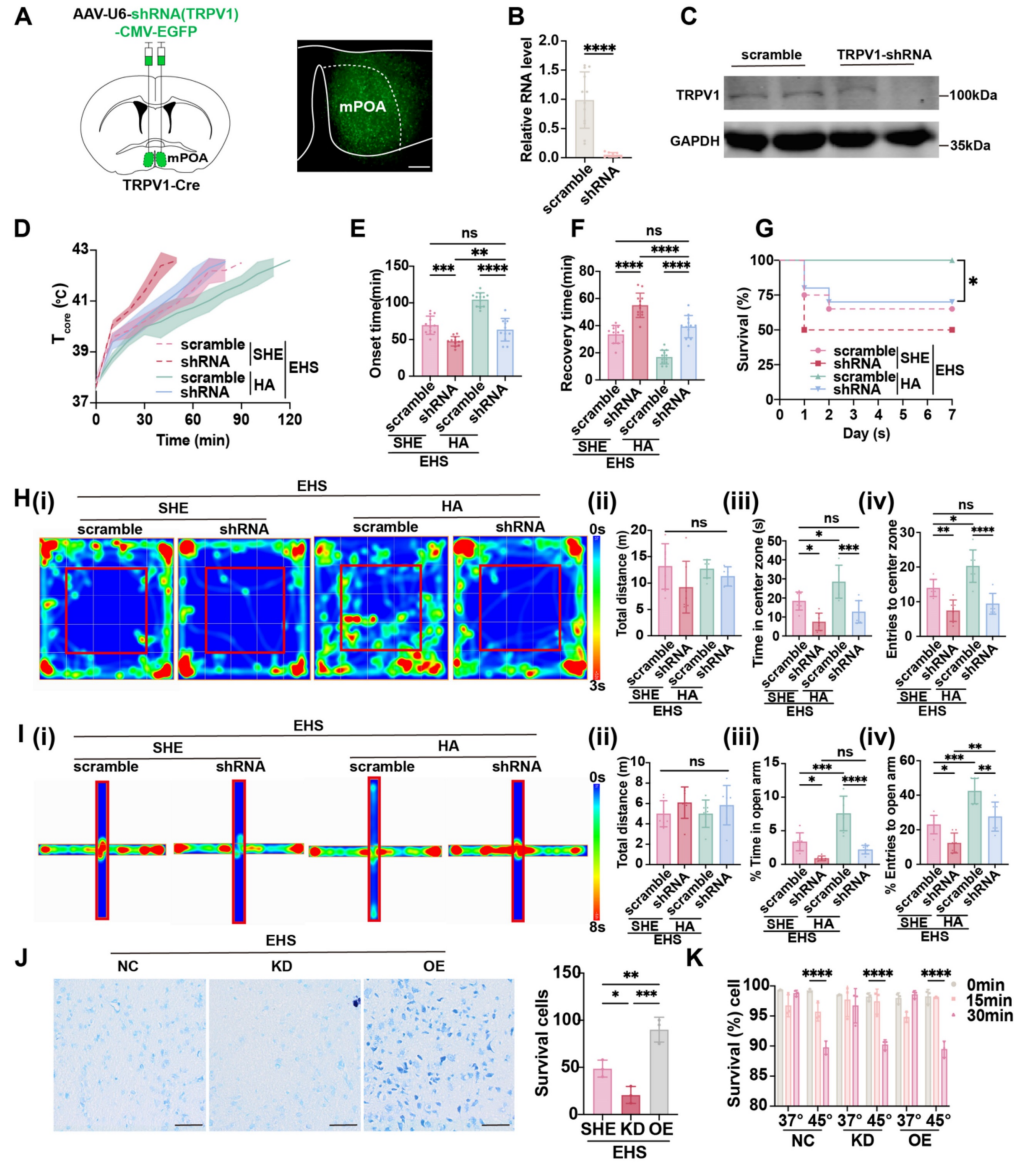
** TRPV1 is critical for HA-triggered heat resistance.** (A) Scheme and representative coronal images of the injection of AAV-U6-shRNA (TRPV1)-CMV-EYFP into mPOA. Scale bar, 100 µm. (B-C) The validation of knockdown efficiency by qPCR and western blot. (D) The changes of *T*core during the modeling process. (E-F) The onset time of heat-syncope (E) and recovery time post EHS (F) (n = 12). (G) 7-day mortality rate in each group of mice (n = 20). (H) Typical tracking chart of the OFT (i), statistics of total distance travelled (ii), time spent in the center zone (iii), and the number of entries to the center zone (iv) in the OFT at 48 h of recovery (n = 7). (I) Typical tracking chart of the EPM (i), statistics of total distance travelled (ii), time spent in the open arm (iii), and the number of entries to the open arm (iv) in the EPM at 48 h of recovery (n = 7). (J) Nissl staining experiments in mPOA area in the SHE group, TRPV1-overexpress (TRPV1-OE) group, and TRPV1-shRNA (TRPV1-KD) group (n = 3). Scale bar, 100 μm. (K) Trypan Blue staining experiments in Neuro-2a cells (NC/ TRPV1-OE/ TRPV1-KD) to examine cell survival rates at 0 min, 15 min, and 30 min after heat stress (45 °C) and control temperature (37 °C) (n = 3). All data are shown as mean ± SD and were analyzed by one-way ANOVA, or two-way ANOVA. *p < 0.05, **p < 0.01, ***p < 0.001, ****p < 0.0001, ns, not significant.

Considering that the expression of TRPV1 was enhanced by HA training (Figure 2B and 2C) and preoptic TRPV1 neurons functioned as warm-sensitive neurons (Figure 4 and 5), we hypothesized that TRPV1 acts as not only a cell marker but also a functional molecule. The AAV carrying shRNA for *Trpv1* mRNA (rAAV-U6-shRNA (TRPV1)-CMA-EGFP) was injected into mPOA (Figure 6A), with scramble shRNA as control, and the knockdown efficiency of TRPV1 was confirmed both at RNA level by qPCR (Figure 6B) and protein level by western blot (Figure 6C). Under the EHS circumstances, silencing TRPV1 would accelerate the *T*_core_ elevate (Figure 6D), shorten the onset time of heat-syncope (47.67 ± 12.46 min vs 69.50 ± 12.46 min with control, *p* = 0.0002, Figure 6E), and prolonged the recovery time (55.00 ± 8.985 min vs 33.58 ± 6.598 min with control, *p* < 0.0001, Figure 6F). The mortality rate also deteriorated in the TRPV1 knockdown group (Figure 6G). The total distance travelled in OFM remained unchanged of different groups, while both time and number of entries to the center zone significantly collapsed in the TRPV1 knockdown group (Figure 6H). Consistently, both the time in and entries to the open arm of the TRPV1 knockdown mice decreased compared with the scramble group in the EPM tests (Figure 6I). These results indicated that TRPV1 acted as a functional molecule for the activation of warm-sensitive neurons in mPOA, the downregulation of which would affect the animal heat defense system. Moreover, although HA training could enhance the *T*_core_ regulation process and alleviate the EHS, silencing TRPV1 in mPOA neurons could reverse the protective effects of HA pretreatment (Figure 6D-6G). Additionally, the improvement of neurological prognosis post EHS by HA training, as detected by OFM and EPM, was also blocked in the TRPV1 knockdown group (Figure 6H and 6I).

Considering the limitation of knockdown efficiency, TRPV1 knockout (KO) was used to further elucidate the role of TRPV1 in our study. TRPV1 KO mice were identified as positive by PCR screening (Figure S3A). Under EHS exposure, TRPV1 KO mice subjected to HA training exhibited a hastened increase in core body temperature (*T*_core_) (Figure S3B), a reduced latency to the onset of heat syncope (59.08 ± 10.77 min vs 113.6 ± 12.94 min with control, *p* < 0.0001, Figure S3C), and an extended recovery time (27.00 ± 9.496 min vs 17.67 ± 7.365 min with control, *p* = 0.0134, Figure S3D). Moreover, the mortality rate was significantly higher in the TRPV1 KO+HA+EHS group (Figure S3E). In the OFM, the total distance traveled did not vary among groups; however, the time spent and entries to the center zone were markedly reduced in the TRPV1 KO+HA+EHS group (Figure S3F). In concordance, both the duration and frequency of entries to the open arm were diminished in TRPV1 KO+HA+EHS mice compared to the wild type (WT)+HA+EHS group in the EPM test (Figure S3G).

Given the distribution and functional implications of TRPV1 in both peripheral and spinal cord tissues, we sought to ascertain its role in the brain using TRPV1^floxed^ mice. Our findings indicate that the selective ablation of TRPV1 in the brain also accelerated the elevation of *T*_core_ (Figure S4B), decreased the latency to heat syncope onset (71.42 ± 13.11 min vs 94.75 ± 10.12 min with control, *p* < 0.0001, Figure S4C), and prolonged the recovery time (33.58 ± 10.34 min vs 20.92 ± 6.585 min with control, *p* = 0.0017, Figure S4D). The mortality rate was also exacerbated in the TRPV1 flox+HA+EHS group (Figure S4E). In the OFM, no significant difference in total distance traveled was observed; however, the time and number of entries to the center zone were significantly reduced in the TRPV1 flox+HA+EHS group (Figure S4F). Consistently, the time spent and entries to the open arm in the EPM test were decreased in TRPV1 flox+HA+EHS mice compared to the scramble control group (Figure S4G). These results indicated that TRPV1 acted as a functional molecule for the activation of warm-sensitive neurons in mPOA, the downregulation of which would affect the animal heat defense system.

It has been reported that EHS could trigger neuron death in hypothalamus 3. To assess whether preoptic TRPV1 expression exerted heat resistance effects by influencing the neuron activity or by promoting neuronal survival against EHS, both *in vivo* and *in vitro* experiments were performed. We found that the overexpression of TRPV1 did alleviate the EHS-induced neuron death of mPOA as detected by Nissl staining, the knockdown of which aggravated neuronal loss post EHS (Figure 6J). However, intervening TRPV1 in Neuro-2a cells, either overexpressing of silencing TRPV1 expression, could not alter the heat induced cell death (Figure 6K). This suggested that TRPV1 might enhance heat tolerance by modulating the function of mPOA^TRPV1^ warm-sensitive neurons but not by directly promoting neuronal survival. Taken together, TRPV1 was critical for HA-triggered heat resistance by facilitating the activation of warm-sensitive neurons in mPOA.

### The myokine irisin might improve neuron function by promoting TRPV1 expression

**Figure 7:**
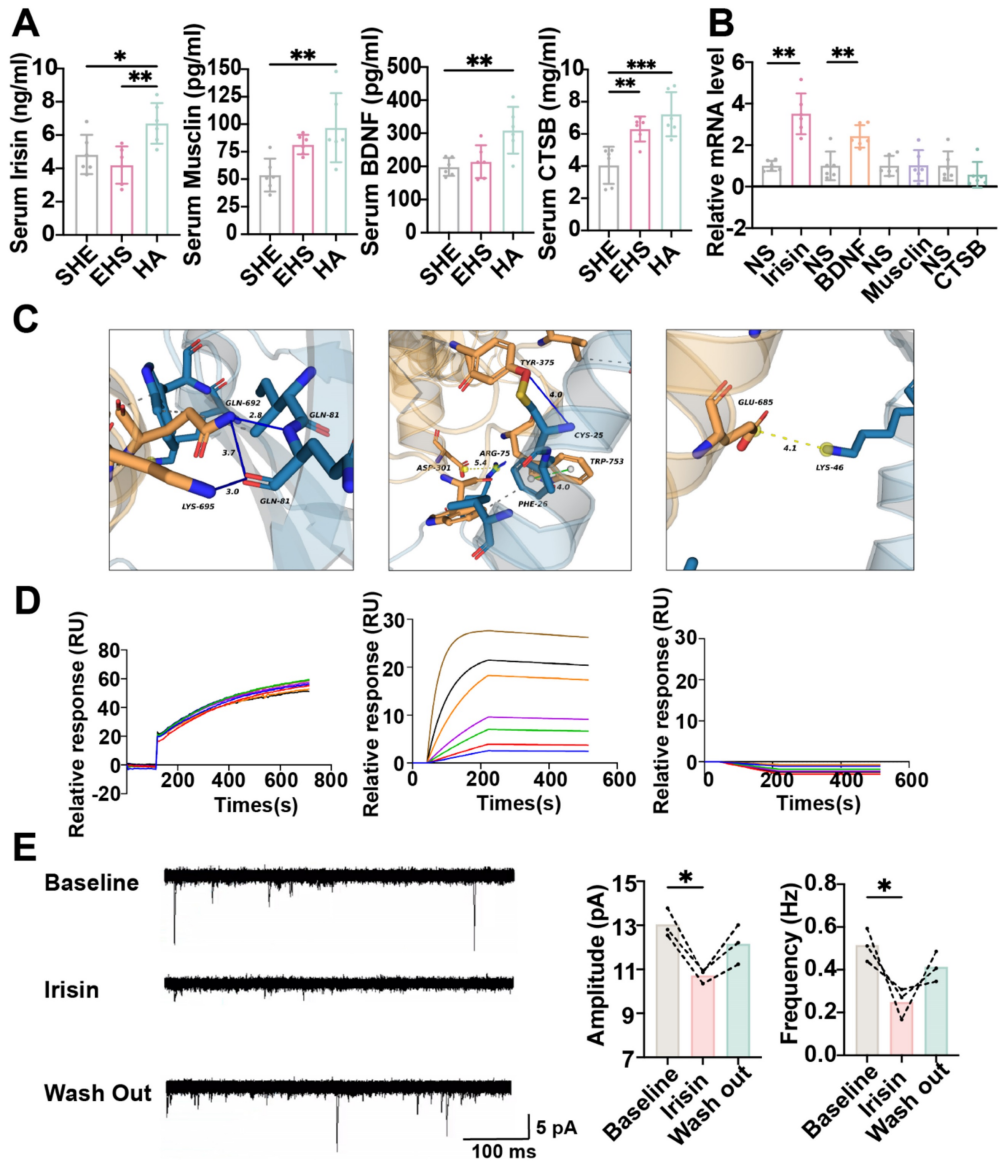
** Irisin improves neuron function by promoting TRPV1 expression.** (A) Result of ELISA assay (n = 6). (B) The result of relative TRPV1 mRNA level (n = 3). (C) The results of molecular docking analysis. (D) The results of surface plasmon resonance. (E) Schematic illustration/analysis of EPSC of mPOA^TRPV1^ neurons (n = 3). All data are shown as mean ± SD and were analyzed by one-way ANOVA, or two-way ANOVA. *p < 0.05, **p < 0.01, ***p < 0.001, ****p < 0.0001, ns, not significant.

To investigate the mechanism of how HA training promoted the preoptic TRPV1 expression, we first detected muscle-derived neuroprotective myokines as previously reported, including irisin, musclin, BDNF and CTSB under different conditions 31,34,52-55. Compared with the SHE control group and the EHS exposure group, the serum concentration of irisin and CTSB significantly increased in the HA training group (Figure 7A). Then these myokines were injected to the mPOA with the stereotaxic apparatus, and the *Trpv1* mRNA level in mPOA was assessed by qPCR. It was irisin and BDNF, but not musclin and CTSB, that could strengthen TRPV1 expression (Figure 7B). To further detect whether irisin could target and activate TRPV1 directly besides enhancing *Trpv1* transcription, further bioinformatic analysis surface plasmon resonance (SPR) experiments and patch clamp assays were performed. Molecular docking analysis indicated that multiple amino acid residues in the TYR-375, TRP-753, LYS-692, and LYS-695 could interact with irisin (Figure 7C). SPR experiments revealed that the affinity constant between TRPV1-nanodisc immobilized on the LifeDisc™ lipid (LB) biosensor and irisin protein was 10.2 nM (Figure 7D). Meanwhile, there is no specific binding between biosensors and irisin (Figure 7D). These results suggested that irisin might bind to TRPV1.

Hence, we hypothesized that irisin stimulation might increase the frequency and amplitude of EPSCs for mPOA^TPRV1^ neurons, thus improving their function with EHS exposure. Unexpectedly, electrophysiological data showed that irisin treatment resulted in decreases in the frequency and amplitude of EPSCs (Figure 7E). Furthermore, we observed that a single administration of irisin prior to EHS modeling did not confer significant protective effects (Figure S5). These findings could not support the hypothesis that irisin enhances heat resistance through the direct activation of TRPV1 receptors.

Considering that irisin may exert its protective influence by upregulating TRPV1 expression in response to repeated heat challenges, irisin protein was intraperitoneally injected repeatedly for seven days in WT or TRPV1 KO mice. In WT mice, irisin treatment before EHS modeling was found to augment the resting membrane potential and diminish the rheobase of mPOA^TRPV1^ neurons when compared to the SHE group (Figure S6A-S6B). Furthermore, the frequency and amplitude of EPSCs and the number of AP in mPOA^TRPV1^ neurons were increased in the irisin-pretreated EHS group relative to the SHE group (Figure S6C-S6D). Conversely, no significant differences were observed between the EHS and SHE groups in irisin-pretreated TRPV1 KO mice. Collectively, these indicated that irisin might improve the function of POA^TRPV1^ neurons not by directly activating them. In brief, the irisin originated for HA training possibly improving neuron function by promoting TRPV1 expression.

### Exosomes specially delivering irisin to CNS protected mice from EHS

**Figure 8:**
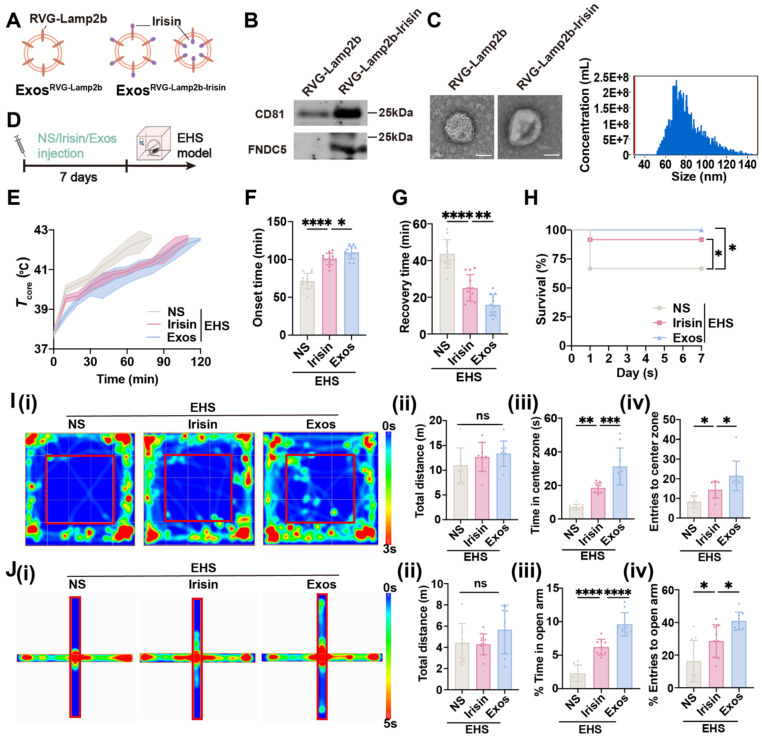
** Irisin simulating HA protects against EHS in mice.** (A) Scheme of plasmid construction. (B) Western blots of exosomes prepared from supernatants of 293T cell culture. (C) Electron microscope images of Exos^RVG-Lamp2b^ and Exos^RVG-Lamp2b-Irisin^. Scale bars, 50 nm. (D) Scheme of injection of different drugs. (E) The changes of *T*core during the modeling process. (F-G) Statistics of the onset time of heat-syncope (F) and recovery time post EHS (G) (n = 12). (H) Statistics of the 7-day mortality rate in each group of mice (n = 12). (I) Typical tracking chart of the OFT (i), statistics of total distance travelled (ii), time spent in the center zone (iii), and the number of entries to the center zone (iv) in the OFT at 48 h of recovery (n = 9). (J) Typical tracking chart of the EPM (i), statistics of total distance travelled (ii), time spent in the open arm (iii), and the number of entries to the open arm (iv) in the EPM at 48 h of recovery (n = 9). All data are shown as mean ± SD and were analyzed by one-way ANOVA, or two-way ANOVA. *p < 0.05, **p < 0.01, ***p < 0.001, ****p < 0.0001, ns, not significant.

The RVG-lamp2b-Irisin exosome system was applied to efficiently deliver irisin to the brain as previously reported (Figure 8A) 56. The plasmids were exogenously expressed in HEK 293T cells, and the exosomes were isolated in the supernatants by ultracentrifugation. The acquired exosomes were identified by immunoblot for exosome marker CD81 and target protein FNDC5/irisin (Figure 8B), as well as by transmission electron microscope to confirm their diameter (40-100 nm) (Figure 8C). To elucidate the protective effects of exosomes delivering irisin (Exos), the normal saline (NS) and original irisin polypeptides were used as control, which were all intraperitoneally injected before EHS modeling (Figure 8D). As expected, both irisin polypeptides and Exos pretreatment could delayed the *T*_core_ elevation with EHS environment (Figure 8E). Intriguingly, Exos group exhibited better heat tolerance ability than the irisin polypeptide group, which maintained late onset time of heat-syncope (109.3 ± 8.226 min , vs 100.5 ± 7.574 min with the irisin polypeptide group, *p* = 0.0371; 109.3 ± 8.226 min vs 71.33 ± 10.28 min with the NS group, *p* < 0.0001; Figure 8F) and prompt recovery time (15.83 ± 5.573 min vs 25.08 ± 7.192 min with irisin polypeptide group, *p* = 0.0045; 15.83 ± 5.573 min vs 43.67 ± 7.691 min with NS group, *p* < 0.0001; Figure 8G). The srvival rate was also significantly improved in the Exos group (Figure 8H). Moreover, Exos pretreatment showed a higher increase in both time and number of entries to the center zone than the irisin polypeptide group for the OFM tests (Figure 8I). Consistently, both the time in and entries to the open arm of EHS mice increased in the Exos group compared with the irisin polypeptide group for EPM tests (Figure 8J). Taken together, these data indicated that Exos pretreatment exhibited better preventive effects than the administration of irisin polypeptides against EHS.

### Irisin upregulates TRPV1 expression via PI3K/AKT pathway

**Figure 9:**
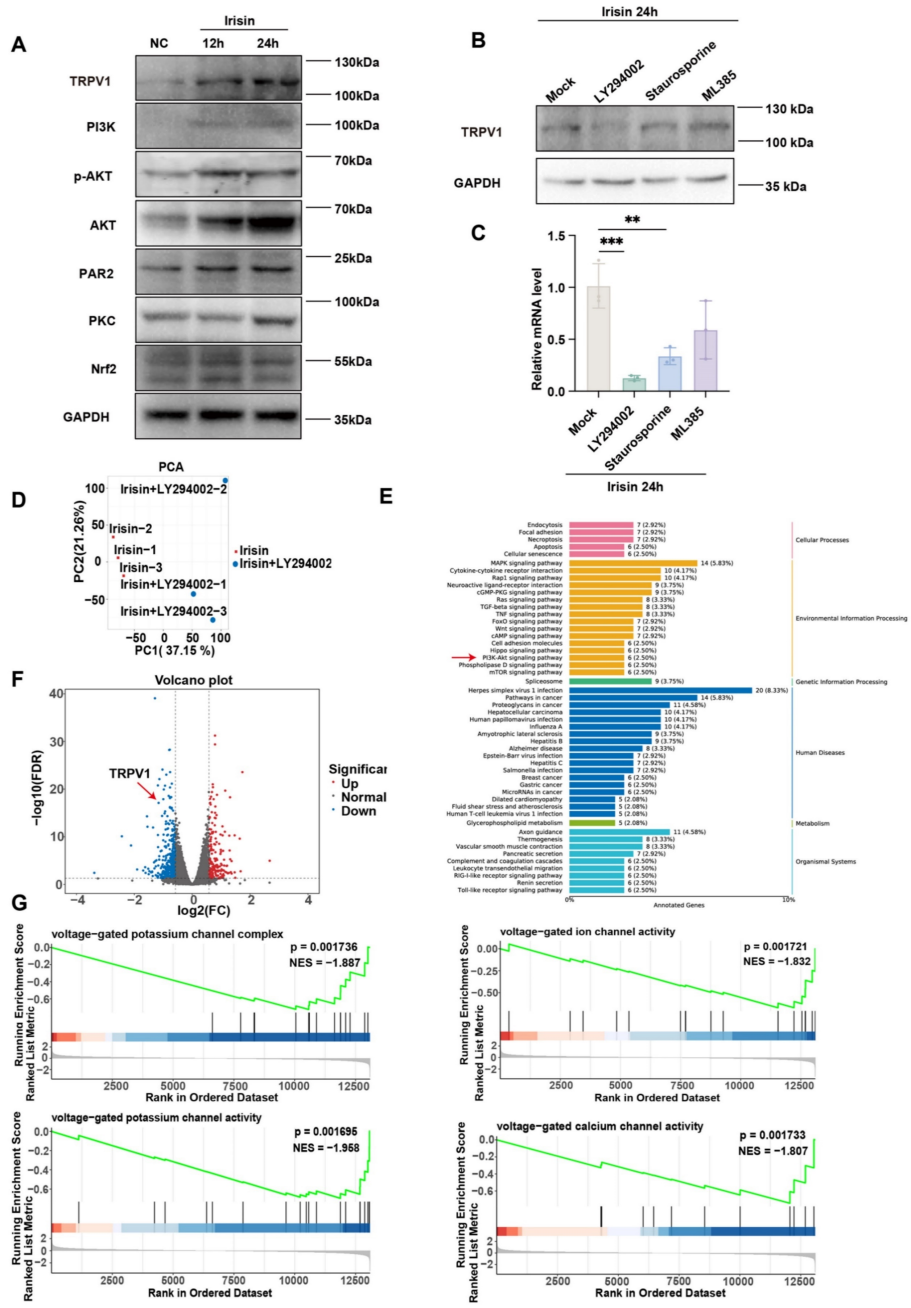
** Irisin upregulates TRPV1 expression via PI3K/AKT pathway.** (A) The expression levels of TRPV1, PI3K, AKT, p-AKT, PAR2, PKC, and Nrf2 were measured by western blot. (B-C) The expression level of TRPV1 in groups pretreated with irisin and different inhibitors was measured by western blot (B) and qPCR (n = 3) (C). (D) The result of principal component analysis (PCA) between irisin and irisin+LY294002 groups. (E) Gene expression difference volcano diagram. Each point in the graph represents a gene, with red indicating upregulated genes, green indicating downregulated genes, and black indicating non- differentially expressed genes. TRPV1 as indicated by the red arrow. (F) Results of the Kyoto Encyclopedia of Genes and Genomes (KEGG) analysis. (G) Results of Gene Set Enrichment Analysis (GSEA).

Previous articles suggest that irisin may regulate downstream activation via the PI3K/AKT 57-59, PAR2/PKC 60-62, and Nrf2/GPX4 63-65 pathways. To empirically ascertain which pathway makes sense, we detected their activation by immunoblot with or without irisin. The expression levels of TRPV1, PI3K, AKT, phospho-AKT (p-AKT), PAR2, PKC, and Nrf2 significantly elevated in Neuro-2a cells subsequent to irisin stimulation, thereby corroborating the activation of the aforementioned signaling cascades by irisin (Figure 9A). Next, the expression of TRPV1 was detected post irisin stimulation with or without different inhibitors, which included the AKT inhibitor LY294002, the PKC inhibitor Staurosporine, and the Nrf2 inhibitor ML385. It was found that although each inhibitor attenuated TRPV1 expression, the LY294002 exerted the most pronounced suppressive effects, both in protein (Figure 9B) and mRNA level (Figure 9C).

To substantiate our results, RNA-seq was performed in LY294002-treated Neuro-2a cells post irisin stimulation. Principal Component Analysis (PCA) between irisin and irisin+LY294002 groups was shown in Figure 9D. KEGG analysis revealed that the PI3K/AKT pathway was enriched (Figure 9E). The results indicated that TRPV1 expression was downregulated after LY294002 pretreatment (Figure 9F). Meanwhile, Gene Set Enrichment Analysis (GSEA) also revealed that the enriched pathways in the irisin+LY294002 group mainly downregulated the activation of voltage-gated potassium channels and voltage-gated calcium channels (Figure 9G). These results suggest that irisin may upregulate TRPV1 expression through the activation of the PI3K/AKT pathway.

## Discussion

**Figure 10:**
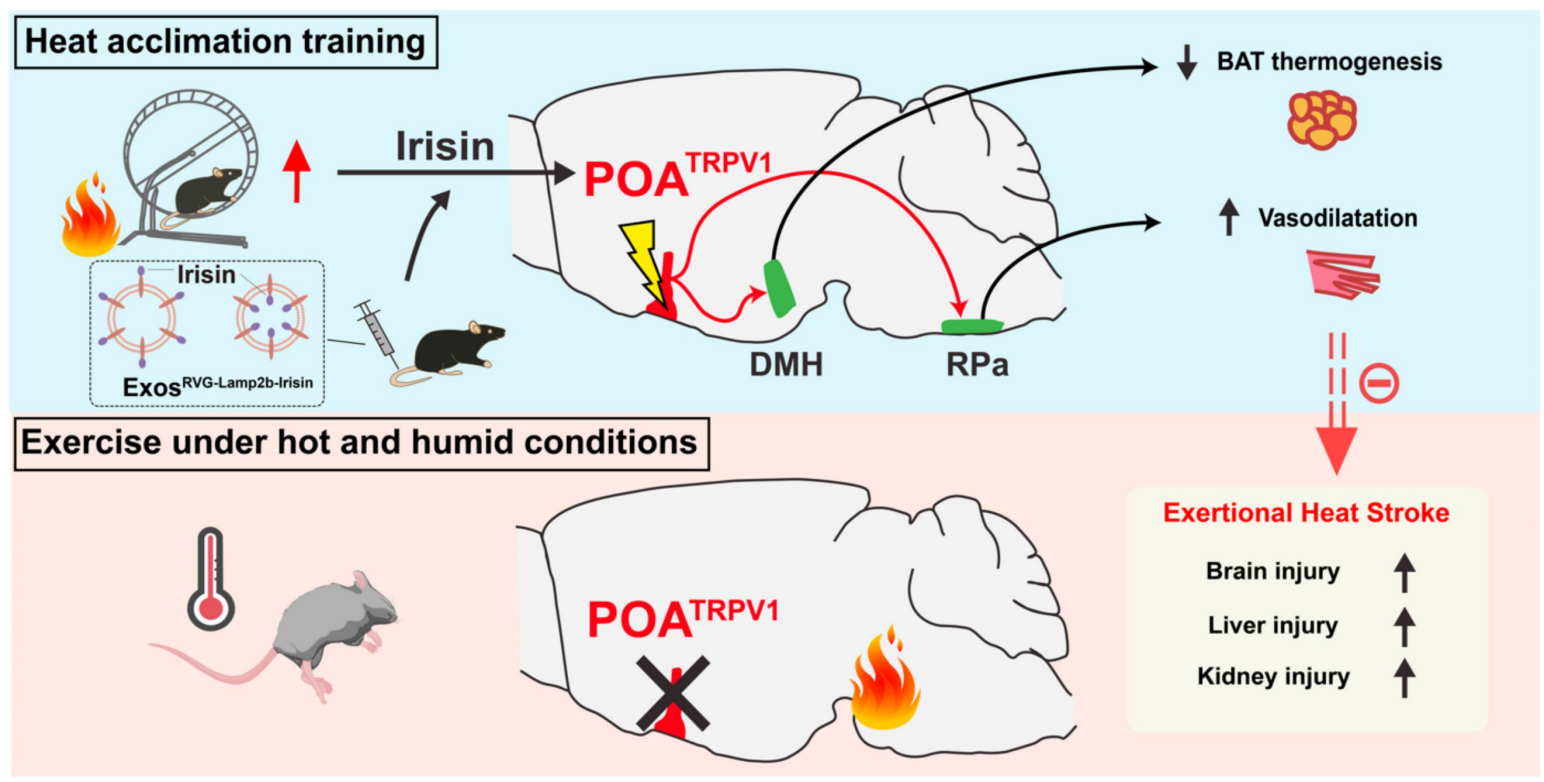
** HA training defense against EHS by improving the function of preoptic TRPV1 neurons.** Heat acclimation might enhance the function of mPOA^TRPV1^ neurons by secreting irisin and promoting TRPV1 expression. The preoptic TRPV1 neurons defend body temperature elevation through the mPOA → DMH/RPa circuit, suppressing iBAT thermogenesis and facilitating vasodilatation, respectively. A novel exosome strategy delivering irisin to CNS could exert protective effects against exercise heat stroke.

It has been predicted that heat waves and high temperatures will be more frequent than ever with worsened global climate changes, which is associated with a rapid increase in the morbidity and mortality of heat stroke 66-68. Heat stroke is involved with multiple organ dysfunction, which includes liver, lung, intestine, hippocampus and corpus striatum 3,69,70. Although previous investigations have demonstrated that the pathogenesis of hyperthermia-induced tissue injury is mainly caused by cellular necroptosis 3 or other programmed cell death processes 69,71, metabolism disruption 70,72, oxidative stress 73, mitochondrial dysfunction 66 and other molecular mechanisms, it still remains obscure about how the neuronal circuits that control thermoregulatory process are affected during heat defense against heat stroke, especially EHS. Here, we report that the preoptic TRPV1 neurons defend body temperature elevation through the mPOA" DMH/RPa circuit, which results in the suppression of iBAT thermogenesis and facilitation of vasodilatation. HA training enhances the function of mPOA^TRPV1^ neurons by secreting irisin and promoting TRPV1 expression, strengthening heat defense and ameliorating EHS-related injuries. Moreover, a novel strategy (RVG-lamp2b-Irisin) delivering irisin to CNS is constructed, which exerts protective effects against EHS (Figure 10).

Our results revealed a new sight to explicate why the TRPV1 antagonists that were used as analgesics either in animal experiments or preclinical trials (such as AMG517 74, ABT-102 75, AZD1386 76, and JNJ-39439335 77) could produce the undesirable hyperthermic effects. TRPV1, known for its sensitivity to pungent vanilloid compound (i.e. capsaicin), is a non-selective cation channel activated by diverse stimuli, including acidosis, noxious heat, toxins, and various endogenous or exogenous ligands (such as inflammatory mediators) 41,78,79. Preceding researches primarily concentrated on the role of TRPV1 as nociceptor which mediates the neuropathic pain and bridges the neuroimmune interactions 80,81. On the one hand, TRPV1 was mainly distributed in the sensory neurons, including dorsal root neurons, trigeminal neurons, and small and medium-sized neurons of the vagal ganglion 82. Stimulation of TRPV1 evoked a burning sensation that transferred the noxious physical or chemical stimuli to electrical signals, and reflected a central role of the channel in pain 79,83. Pharmacological or genetic intervention investigations validated TRPV1 as a therapeutic target for analgesics, the antagonists of which were proved to be effective for controlling the clinical chronic pain (i.e. cancer, neuropathic, postoperative or musculoskeletal pain) 79. On the other hand, TRPV1 was also expressed in microglia, which exerted either synergistic or adverse effects on neuroinflammatory responses 84. TRPV1 promoted the activation of ATP-induced NLRP3 inflammasome by mediating Ca^2+^ influx and phosphorylation of phosphatase PP2A in microglia, the ablation of which reduced the neuroinflammation and alleviated mice experimental autoimmune encephalomyelitis 85 as well as the brain injury post subarachnoid hemorrhage 86. Activating of TRPV1 channels on microglia can boost autophagy for clearance of α-Synuclein and improve the treatment of Parkinson's Disease 87. In contrast, several researches showed that TRPV1 could suppress the inflammatory reactions and synaptic phagocytosis by inhibiting lipid accumulation of microglia in neurodegenerative diseases 88,89, and could also restrain the NLRP3 activation in microglia by regulating autophagy after ischemia-reperfusion injury 90. To note, pharmacological modulation of TRPV1 for pain management have been limited by the deleterious hypothermia or hyperthermia caused by TRPV1 agonists/antagonists, the mechanisms of which referred to the peripheral actions on the vasculature 91, and the modulation of sensory input to the CNS 92. Recent study showed that it was the biased allosteric alteration of TRPV1 that determined its analgesic or thermoregulatory effects 93. These researches mainly focused on the peripheral manners, while increasing evidences discovered a new role of TRPV1 in CNS that altered gene expression related to synaptic plasticity by epigenetic regulation 94, and affected contextual fear memory, anxiety and stress-related behavior 95,96. In our study, a group of TRPV1 positive neurons were identified as warm-sensitive neurons in mPOA, which were activated in the EHS environment and the function of which was improved by HA training (Figure 2). Moreover, although the mPOA^TRPV1^ " DMH neurons were mixed both glutamatergic and GABAergic populations, the major projection of which was GABAergic mPOA^TRPV1^ to glutamatergic DMH neurons, restricting the DMH-mediated iBAT thermogenesis (Figure 3). Optogenetic or chemogenetic assays also confirmed the thermoregulatory effects of mPOA^TRPV1^ neurons (Figure 4). This might partially explain why inactivation of TRPV1 neurons for pain alleviation could lead to hyperthermia side effects from the aspect of CNS dysfunction.

Although we found that HA training could strengthen the function the preoptic TRPV1 neurons, the specific mechanism is still opaque. Previous studies suggested that the capsaicin, a canonical agonist for TRPV1, could induce a long-lasting, reversible desensitized state of TRPV1 that cannot be activated secondarily 97, decreasing Tb through facilitating the vasodilation, sweating, and wheezing 93,98. It was worthy noting that HA training could not directly enhance the excitability of mPOA^TRPV1^ neurons as detected by electrophysiological experiments post EHS exposure (Figure 2I-2K) but could make them generate more APs with the identical injected currents (Figure 2L). This suggested that HA training enhanced heat resistance not by increasing the excitability of mPOA^TRPV1^ neurons, but possibly by making them more sensitive to stimulation with more TRPV1 expression. Then, there existed the possibility that TRPV1 might act as a sensor for hyperthermia in the CNS and improve the durability of warm-sensitive neurons in mPOA (which meant that higher TRPV1 expression level might counteract the desensitized state of itself), or that TRPV1 might inhibit the heat-induced neuronal injury. Considering that the *in vitro* experiments demonstrated that either TRPV1 knockdown or overexpression could not improve heat-triggered neuron death (Figure 6K), excluding the latter hypothesis. The activation of TRPV1 in mPOA might be caused by heat, inflammatory mediators, or other signals from peripheral neurons, while further studies should be performed to investigate how mPOA^TRPV1^ neurons were affected with EHS exposure. Taken together, our research showed that TRPV1 represented not only the biomarker but also the functional molecular for warm sensitive neurons in the mPOA.

It has been proved the HA was one of most efficient strategies in preventing EHS 99,100. And in this study, we discovered that it was the irisin produced during HA training that intensified heat resistance. Irisin, a muscle-secreted hormone, was generated by cleavage of membrane protein FNDC5 and acted as a mediator of exercise-induced metabolic improvements by browning of white adipose tissue 101. Irisin might be an ideal therapeutic target for not only the metabolic disease by reducing diet-induced obesity and insulin resistance 35, but also the non-metabolic diseases including sarcopenia 102, osteoporosis 103. Although recent researches indicated that irisin could improve the cerebral cognitive function by rescuing synaptic plasticity and activating autophagy in neurodegenerative diseases, the thermoregulatory role of irisin has long been ambiguous. Several studies suggested that irisin increased in energy expenditure by stimulating UCP1 expression and a broad program of brown-fat-like development, which might help maintain core temperature with mild cold exposure 35,104. In our study, the results of molecular docking and SPR showed that there is an interaction between irisin and TRPV1. Intriguingly, in patch clamp or *in vivo* experiments, we found that repeated irisin stimulation can stimulate TRPV1 mediated heat resistance, rather than direct stimulation (Figure 7, S5 and S6). These suggested that irisin mimicked the protective effects of HA training by promoting TRPV1 expression in mPOA neurons in HA training but not directly binding to and priming them. It has been reported that irisin could activate multiple survive or stress associated signals, such as PI3K/AKT 105, PAR2/PKC 62, Nrf2/GPX4 106 pathways. Our results found that TRPV1 expression along with the activation of voltage-gated potassium channels and voltage-gated calcium channels were downregulated after AKT inhibitor LY294002 pretreating (Figure 9). This suggests that irisin may upregulate TRPV1 expression through the activation of the PI3K/AKT pathway.

Moreover, although irisin itself could traverse across the blood-brain barrier, we also confirmed that the exosomes (RVG-lamp2b-Irisin) containing RVG and irisin showed better neuroprotective effects against EHS than merely irisin administration (Figure 8), which might be involved that exosomes delivered more irisin to the CNS.

In summary, we demonstrated that HA defense against HE by improving the function of preoptic TRPV1 neurons, which was involved with the irisin-induced expression of TRPV1 in mPOA. The mPOA^TRPV1^ " DMH/RPa circuit played a significant role in heat defense by restricting iBAT thermogenesis and facilitating vasodilatation, and this process could be strengthened by HA straining or application of exosomes delivering irisin.

## Materials and methods

### Animal

This part of the experiment was approved for implementation by the Animal Ethics Committee of the Air Force Military Medical University. The animals used were wild-type C57BL/6 mice aged about 2 months and weighing 20-25 g, male. Mice were housed with a 7:00 am-7:00 pm light and a 7:00 pm-7:00 am^+1^ darkness cycle. Mice were singly housed under controlled temperature (25 ± 2 °C) and constant humidity (40 ± 10 % RH). The transgenic mice TRPV1-IRES-Cre and TRPV1^floxed^ used in this study were bred by Shanghai Model Organisms Center. TRPV1 KO mice used in this study were bred by Cyagen.

### Separate heat exposure

The mice were placed in a constant temperature chamber at 35 ± 1 °C, 70 ± 5 % RH to receive heat exposure. Maintain the same daily training period. The training time is 1 h per day for a total of 7 days.

### Heat acclimation training

We forced the running wheels to be placed in a constant temperature chamber at 35 ± 1 °C, 70 ± 5 % RH, allowing the mice to exercise in a warm environment. Maintain the same daily training period. The training time is 1 h per day for a total of 14 days. The speed gradually increases compared to before regular training and remains constant after increasing to 15 r/min.

### Exertional heat stroke modeling

The mice were exposed to constant temperature in a constant temperature chamber. To avoid discomfort caused by gas blowing directly onto the experimental mice, the fan window is designed above the cabin to create air convection. The mice in the cabin showed no significant discomfort. The constant temperature cabin is covered with organic glass panels, and gaps are left for easy light transmission, observation of animal behavior, and subsequent optical fiber transmission for calcium signal recording experiments.

Modeling: First, place the forced runner in a constant temperature environment chamber, and maintain stability after increasing the ambient temperature and relative humidity to the target values (38 ± 1 °C, 70 ± 5 % RH). Next, the mice were placed at room temperature to rest until the core temperature dropped to 37.5 °C for 15 min. Then, they were placed on a rotating wheel, and a forced rotating wheel scheme was initiated (at a speed of 13 r/min). Use an infinite remote sensing system to record the core body temperature of mice every 2 min and continuously measure it until it reaches 42.5 °C. Take the mice out of the environmental compartment and place them at room temperature for recovery. Observe the mice until they regain consciousness while continuing to monitor their core body temperature and record their survival status. During the recovery period, the laboratory maintained a 12 h light and dark cycle.

### Serum sample extraction

After administering pentobarbital sodium anesthesia to the mice, the eyeballs were carefully extracted, and blood specimens were collected utilizing a single-use blood collection receptacle. Approximately 800-1000 μL of whole blood was taken from each mouse. After setting at room temperature for 2 h, the blood sample is placed in a low-temperature centrifuge and centrifuged at 3000 r/min for 20 min. The supernatant was meticulously aspirated using a pipette. To ensure measurement accuracy, samples with hemolysis (the supernatant turns red after centrifugation) must be discarded.

### Intravenous injection

The mouse was gently secured within a restrainer, ensuring the full exposure of its tail. The tail was immersed in warm water at 50 °C for a few minutes or repeatedly wiped the tail with a 75 % alcohol cotton ball to achieve disinfection, dilation of blood vessels in the tail, and softening of the epidermal keratin layer. The middle of the tail was positioned over a light bulb, identifying the veins on both sides of the tail. And the thicker and straighter veins were selected for injection. The tail was stabilized with the left hand while the right-hand grasps a meticulously chosen No. 4 needle attached to a syringe so that the needle was parallel to the vein (less than 30 °). Inject the needle from the lower 1/4 of the tail to inject irisin, with an injection volume of 0.15-0.2 μL/g body weight. After pulling out the needle, use a cotton swab to press the injection site gently for 1-2 min to prevent bleeding. The injection time is 10 am, once daily, lasting 7 days.

### Body temperature measurement

In our study, a probe-type anal thermometer, a body temperature implant, and an infrared thermal imager were used to detect body temperature. The probe-type rectal temperature measuring instrument can be used to measure the rectal temperature of mice, which is easy to operate and has accurate numerical values; the body temperature is implanted into the abdominal cavity to measure the core temperature of mice, which can achieve real-time continuous recording; the infrared thermal imager can record and dynamically monitor the ambient temperature, the temperature of the iBAT of the mouse's shoulder blade and the skin temperature of the tail surface in real-time.

VitalView Data Acquisition System Series 4000 (Starr Life Sciences Corp., Oakmont, USA) is used for wireless remote sensing of core body temperature, recorded every 2 min. To record the core temperature, we used a transmitter implanted into the peritoneum (Mini Mitter, Oregon, USA). The mice were placed in a temperature-controlled room in the home cage of the receiver (Mini Mitter, Oregon, USA) at least three days before the experiment. All measurements were taken during the dark phase.

### iBAT and tail skin temperature measurement

Use a thermal infrared camera (A655sc, FLIR) to measure the surface temperature (*T*_iBAT_) of interscapular brown adipose tissue (iBAT) and the temperature of tail skin (*T*_tail_). Infrared images were recorded and analyzed by analyzeZ tool software. To measure the surface temperature of iBAT, shave off the hair on top of the iBAT one day before measurement. *T*_iBAT_ is defined as the average temperature of the area between capillaries. The *T*_tail_ refers to the temperature at the bottom of the tail. We used factory settings to convert the original pixel count into temperature units and analyzed photos taken from the same angle.

### Open field test

The open field box is made of plastic sheets made of acrylic material (50 cm × 50 cm × 50 cm). At the beginning of the experiment, each mouse was placed in a predetermined corner of the box, allowing it to acclimate and freely explore its surroundings for a duration of 5 min. After the experiment, clean the box and thoroughly remove the odor with 75 % alcohol before starting the behavior test of the next mouse. After the end of open field behavior, the autonomous activity and anxiety-like behavior of mice were detected by using Anymaze software. The analysis indicators were the total distance of movement of the mice, and the time and frequency of entering the central area.

### Elevated plus maze

The elevated cross box is made of an acrylic plastic sheet (40 cm × 40 cm × 40 cm), consisting of open arms at both ends (25 cm in length and 5 cm in width), closed arms at both ends (25 cm in length and 5 cm in width), and a central open area (5 cm in length and 5 cm in width), with a height of 50 cm above the ground. At the beginning of the experiment, gently place the mouse from the central area facing the open arm, allowing it to move freely in the box for 5 min before gently retrieved. After the experiment, clean the box and thoroughly remove the odor with 75 % alcohol before starting the behavior test of the next mouse. After the completion of the elevated cross behavior, the anxiety-like behavior of mice was detected by using Anymaze software. The analysis indicators were the proportion of time the mice entered the open arm in the 5 min and the proportion of times they entered the open arm percentage.

### Stereotactic injection

Mice were anesthetized with pentobarbital sodium (80 mg/mL) and removed hair above the skull to fully expose the scalp. Mice were placed in a stereotaxic instrument and used scissors to cut an incision about 1 cm long from the posterior center just above the skull to fully expose the skull. And then hydrogen peroxide was used to fully expose the bone lines and anatomical markers on the skull. Install the injection pump on the stereotaxic device (Rayward) and properly install the injection needle (Hamilton, 65460-05) filled with mineral oil on the injection pump and absorb an appropriate amount of the target virus to be injected. Level the surface of the skull so that the error of the anterior and posterior fontanels does not exceed ± 0.05 mm and the symmetrical position error of the left and right sides does not exceed ± 0.05 mm. According to the reference coordinates, locate the skull above the location of the target nucleus and drill a small opening. The injection needle was inserted into the target site according to the coordinates, such as mPOA (AP: +0.37 mm; ML: ±0.35 mm; DV: -5.20 mm), with an injection rate of 100 nL/min. The injection needle was withdrawn 10 min after the injection. After the experiment was completed, each mouse needed to verify virus expression and confirm its location.

### RNA sequencing

After anesthesia, a slice of the mouse brain was taken in the cerebral sulcus with a thickness of 1mm. Use a 1 mm diameter punch to take out the mPOA. Total RNA was isolated from each replicate (Tiangen RNeasy kit) and poly-A selection was used to remove ribosomal RNA. Sequencing was performed with paired-end 100-base pair reads at an approximate coverage depth of 30 million reads.

Neuro-2a cells were treated with irisin and irisin+LY294002 for 24 h. Wash with PBS and add cell lysis buffer. After the cells are fully dissolved, store them in liquid nitrogen. Set three biological replicates per group. Then we sent the samples to Biomarker Technologies (Beijing, China) for testing.

### Calcium imaging

Two asymmetric bone windows with a diameter of 800-900 m were opened on the surface of the left and right skulls. placing two skull nails. And a skull drill was used to grind the surface of the skull for later dental cement fixation. Following the injection of the AAV2/9-DIO-GCaMP6s virus, the core was placed above the viral injection site. The distance between the end of the plug and the virus injection point is 200-300 μM. After inserting the core, the surface of the skull and the skull nail were covered completely by glue. After the cement solidifies, remove the gripper. Mice were allowed to feed in cages for 3 weeks before the testing began. On the day of recording, the mice fasted for 24 h (8:00 pm-8:00 pm^+1^) and couldn't help but water. Before the test, the equipment was debugged to ensure the connection of the mouse skull top ceramic connector and the optical fiber jumper. Then the mice were kept in a room-temperature environment box and adapted for 10 min before starting the test. After turning on the LED excitation light, the light intensity was adjusted to confirm the stability of baseline. After no obvious drift, the baseline signal was started to record for 20 min. Then the mice were put into the temperature box to record the continuous calcium signal.

### Patch clamp

Patch clamp is used to detect the discharge of mPOA thermosensitive neurons under normal conditions and after perfusion of irisin, to clarify the responsiveness of neurons to irisin. The method is as follows: (1) POA slicing: After anesthesia with isoflurane, brains were removed, and frozen on dry ice. Cold artificial cerebrospinal fluid (ACSF) was used to soak the cross-section, and the dura mater was quickly removed by ophthalmic scissors. The brain was transferred to a circular dish and immersed in cold ACSF. Then the block was repaired and stuck onto the stage mold. Quickly the mold was transferred to a vibration slicer (Leica, Germany), set the parameters of the vibration slicer, and cut the target brain area into slices (thickness: 300 μm); (2) Neuron renaturation: The cut brain slices were transferred to an incubation network at 36.5 °C for 30 min, and then incubated at room temperature for 1-2 h for later use; (3) Transfer the brain slices to the recording bath by perfusion, gently place the platinum wire U-shaped mesh frame on it, and use a peristaltic pump to continuously perfusion pre oxygenated irisin (25 μM) or ACSF, with a flow rate of 1-2 mL/min; (4) EPSC detection: After filling the glass electrode with liquid, fix it and apply positive pressure. After the electrode enters the liquid, provide compensation current. Under the microscope, select cells with clear contours and smooth surfaces for clamping. After forming a high resistance sealing, the negative pressure or high voltage pulse breaks the film. After breaking the membrane, balance for 5 min and wait for the membrane potential and discharge frequency to stabilize before recording. Analyze the data using Clampfit 11.2 and MiniAnyalysis software.

### Immunofluorescence assays

In c-Fos staining, the target area brain slices were immersed in PBS and rinsed three times, each time for 5 min. Then they were blocked for 2 h in 10 % donkey serum. The brain slices were immersed in 0.3 % Triton X-100 to break the membrane and then incubated with 1:1000 Synaptic systems guinea pig polyclonal antibody anti c-Fos (product number 226308) overnight at 4 °C. The brain slices were washed in PBS for 3 times, each time for 10 min. And then they were incubated with secondary antibody (1:500 goat anti-guinea pig 647 antibody (goat anti-rabbit 488, product number: ab150187) from Abcam company) for 1 h and placed in a 37-degree oven. Dilute DAPI at 1:1000 and incubate at room temperature for 10 min. Rinse the brain slices in PBS 3 times for 10 min each time. The brain slices were stuck onto a glass slide, sealed with anti-fluorescence attenuation sealing agent, and observed under a fluorescence microscope.

### qPCR assay

To determine gene expression in mPOA, an RT-qPCR test was conducted. Total RNA was extracted from the samples using RNAiso Plus (TIANGEN, Beijing, China), and then reverse transcribed to cDNA using 5ˣ HiScript III qRT SuperMix (TIANGEN). The PCR was performed using 2ˣChamQ SYBR qPCR Master Mix (TIANGEN) and PCR System (Applied Biosystems, Waltham, MA, USA). The sequences of the primer pairs were as follows:

Pdyn forward, CTCCTCGTGATGCCCTCTAAT;

Pdyn reverse, AGGGAGCAAATCAGGGGGT;

Bdnf forward, TCATACTTCGGTTGCATGAAGG;

Bdnf reverse, ACACCTGGGTAGGCCAAGTT;

Cck forward, AGCGCGATACATCCAGCAG;

Cck reverse, ACGATGGGTATTCGTAGTCCTC;

Adcyap1 forward, ACCATGTGTAGCGGAGCAAG;

Adcyap1 reverse, CCTCGTCTTCTGGTCTGATCC;

Tac1 forward, TTTCTCGTTTCCACTCAACTGTT;

Tac1 reverse, GTCTTCGGGCGATTCTCTGC;

Sst forward, ACCGGGAAACAGGAACTGG;

Sst reverse, TTGCTGGGTTCGAGTTGGC;

Gal forward, GGCAGCGTTATCCTGCTAGG;

Gal reverse, CTGTTCAGGGTCCAACCTCT;

Gad1 forward, CACAGGTCACCCTCGATTTTT;

Gad1 reverse, ACCATCCAACGATCTCTCTCATC;

Trpv1 forward, CCGGCTTTTTGGGAAGGGT;

Trpv1 reverse, GAGACAGGTAGGTCCATCCAC;

Trpv2 forward, CCTCACTGACTCGGCATACAC;

Trpv2 reverse, CCTCGGTAGAACTCATCGGTG;

Trpm2 forward, CTCGGACGCAGGGAAGGTA;

Trpm2 reverse, CTCTTGGACGTGTTTCTTTGGA.

The relative expression of the target genes was normalized to β-actin levels.

### Western blot

Brains were dissected for analysis and extracted the protein by tissue homogenate method. Cell proteins were cracked using RIPA buffer and centrifuged for 30 min. Proteins were quantified by BCA Protein Assay Kit (Beyotime Biotechnology), separated in 10 % SDS-PAGE gels (30 μL/lane), transferred to PVDF membrane, and probed with anti-TRPV1 (1:500, Abcam), anti-PI3K (1: 500, Proteintech), anti-AKT (1: 1000, Proteintech), anti-phospho-AKT (1: 500, Proteintech), anti-PAR2 (1: 500, Proteintech), anti-PKC (1: 1000, Proteintech), anti-NRF2 (1: 500, Proteintech), anti-FNDC5 (1:500, Proteintech), anti-CD81 (1:1000, Abcam), anti-GAPDH (1:1000, Proteintech). The membrane was incubated with secondary antibodies (1:20000, LI-COR) for 1 h, and images were scanned with Odyssey.

### Surface plasmon resonance analysis

The Biacore T200 spectrometer from Cytiva (USA) was utilized for SPR analysis. The preparation of the activator solution involved mixing 400 mM EDC and 100 mM NHS, which was injected into the system. The CM5 sensor chip was then activated with this solution for 420 s at a flow rate of 10 μL/min. The TRPV1 protein (FLP100128, Dima Biotech, China) was diluted to a concentration of 50 μg/mL using an immobilization buffer and injected into the sample channel (Fc3) at a flow rate of 10 μL/min, resulting in typical immobilization levels of 18900 resonance units (RU). The reference channel (Fc1), which did not require ligand immobilization, was deactivated with 1 M ethanolamine hydrochloride at a flow rate of 10 μL/min for 420 s. Irisin was prepared at six different concentrations (160, 80, 40, 20, 10, and 0 μM) in the same analyte buffer. The Fc1-Fc3 channel was perfused with irisin at a rate of 30 μL/min for a 120 s binding phase, followed by a 200 s release phase, all within the analyte buffer. The association and dissociation processes were conducted in the analyte buffer. After each interaction analysis cycle, the sensor chip surface was thoroughly regenerated with a 10 mM glycine-HCl injection buffer at a flow rate of 30 μL/min for 30 s to remove any analyte. The subsequent concentration cycle of analyte irisin involved repeating the injection and regeneration steps. The Biacore T200 evaluation software (Cytiva, USA) was employed to calculate the affinity constant.

### Statistical analysis

All data were statistically analyzed using SPSS 18.0 software. Unpaired t-test was used for comparison between two groups. One-way ANOVA, or two-way ANOVA was used for comparison between the three groups. Paired t-test was used to analyze the within-mouse change from baseline effect between control and experimental interventions. *p* < 0.05 was considered statistically significant, with *p < 0.05, **p < 0.01, ***p < 0.001, ****p < 0.0001; Simultaneously, GraphPad Prism 8 software were used to process the data and plot the results in the form of mean ± standard deviation (mean ± SD).

## Supplementary Material

Supplementary figures.

## Abbreviations

ACSF: artificial cerebrospinal fluid
ALT: alanine aminotransferase
AP: action potential
AST: aspartate aminotransferase
BDNF: brain-derived neurotrophic factor
BUN: blood urea nitrogen
CHS: classic heat stroke
CNS: central nervous system
CTSB: cathepsin B
DREADD: designer receptor exclusively activated by designer drug
DMH: dorsal medial hypothalamus
EHS: exertional heat stroke
EPM: elevated plus-maze
EPSCs: excitatory post-synaptic currents
Exos: exosomes
FNDC5: fibronectin type III domain containing 5
GSEA: gene set enrichment analysis
GO: gene ontology
HA: heat acclimation
Hippo: hippocampus
HS: heat stroke
iBAT: interscapular brown adipose tissue
KEGG: kyoto encyclopedia of genes and genomes
KOG: euKaryotic ortholog groups
mPOA: medial preoptic area
NS: normal saline
OFM: open field maze
PCA: principal component analysis
PGC-1α: peroxisome proliferator-activated receptor-γ coactivator 1α
qPCR: real-time quantitative PCR
RNA-seq: RNA sequencing
RPa: raphe pallidus nucleus
Scr: serum creatinine
SHE: separate heat exposure
SPR: surface plasmon resonance
T_core_: core temperature
T_tail_: surface temperature of tail skin
T_iBAT_: surface temperature of iBAT
TRP: transient receiver potential
TRPV1: transient receptor potential vanilloid-1
WT: wild type
