# Supplementary material for: Heat acclimation defense against exertional heat stroke by improving the function of preoptic TRPV1 neurons
Source: Theranostics. 2025 Jan 1;15(4):1376–98. doi: 10.7150/thno.101422 (PMC11729562; doi:10.7150/thno.101422)
Supplement: Supplementary file 1 — Supplementary figures. [file thnov15p1376s1.pdf]

Supplementary figures

Figure S1

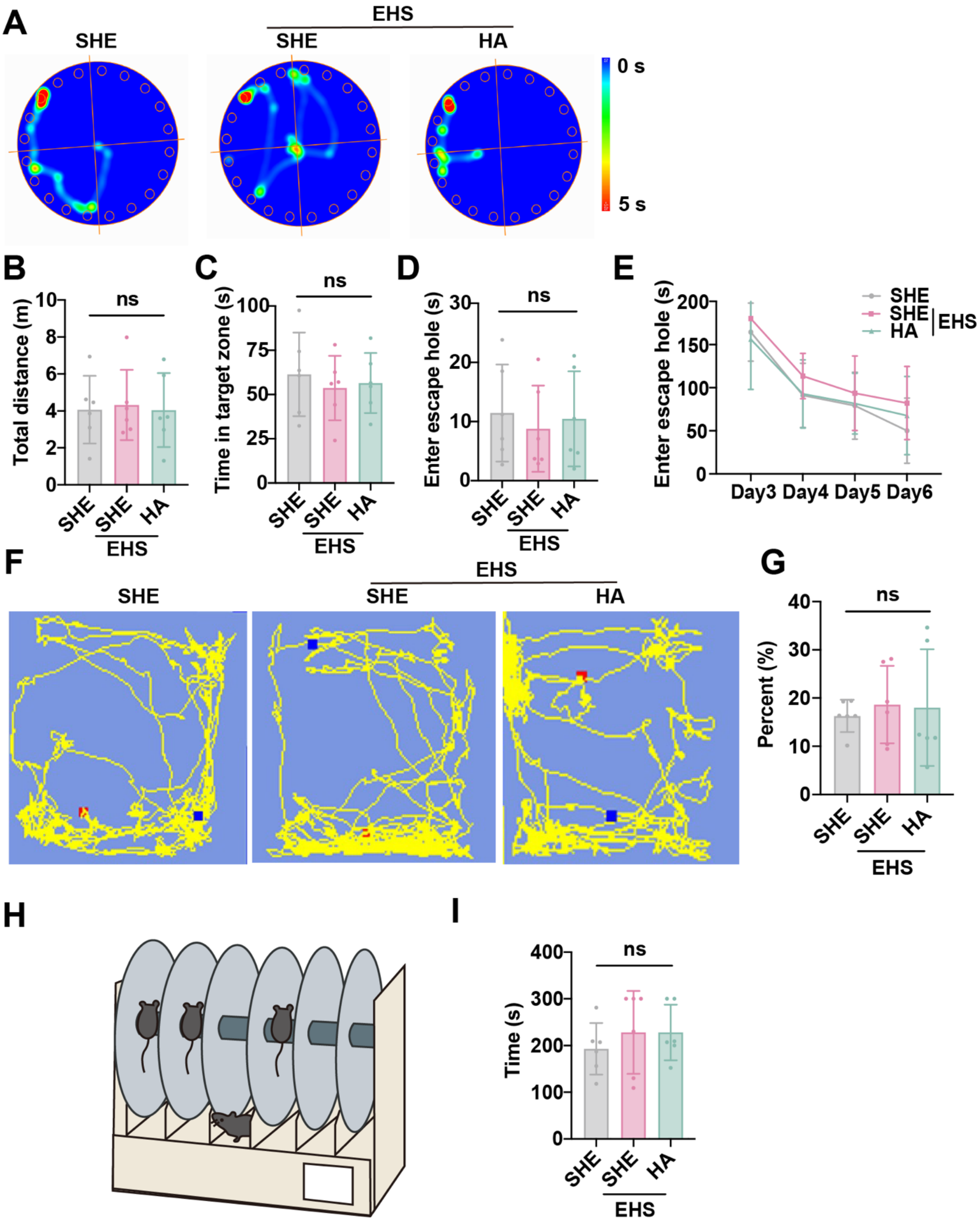

Figure S1. The results of behavior experiment.

(A) Typical tracking chart of the Barnes maze.

(B-D) Statistics of total distance travelled (B), the time in target zone (C), and the latency of enter escape hole (D) in the Barnes maze at 7 days of recovery (n = 6).

(E) Results of the latency of enter escape hole in the Barnes maze during 3-6 days after modeling.

(F) Typical tracking chart of the fear conditioning test at 72 h of recovery.

(G) Statistics of the freezing time in the fear conditioning test at 72 h of recovery (n = 6).

(H) Scheme of the rotarod test at 24 h of recovery.

(I) Statistics of endurance time in the rotarod test at 24 h of recovery (n = 6).

All data are shown as mean ± SD and were analyzed by one-way ANOVA, or two-way ANOVA. *$p < 0.05$, **$p < 0.01$, ***$p < 0.001$, ****$p < 0.0001$, ns, not significant.

Figure S2

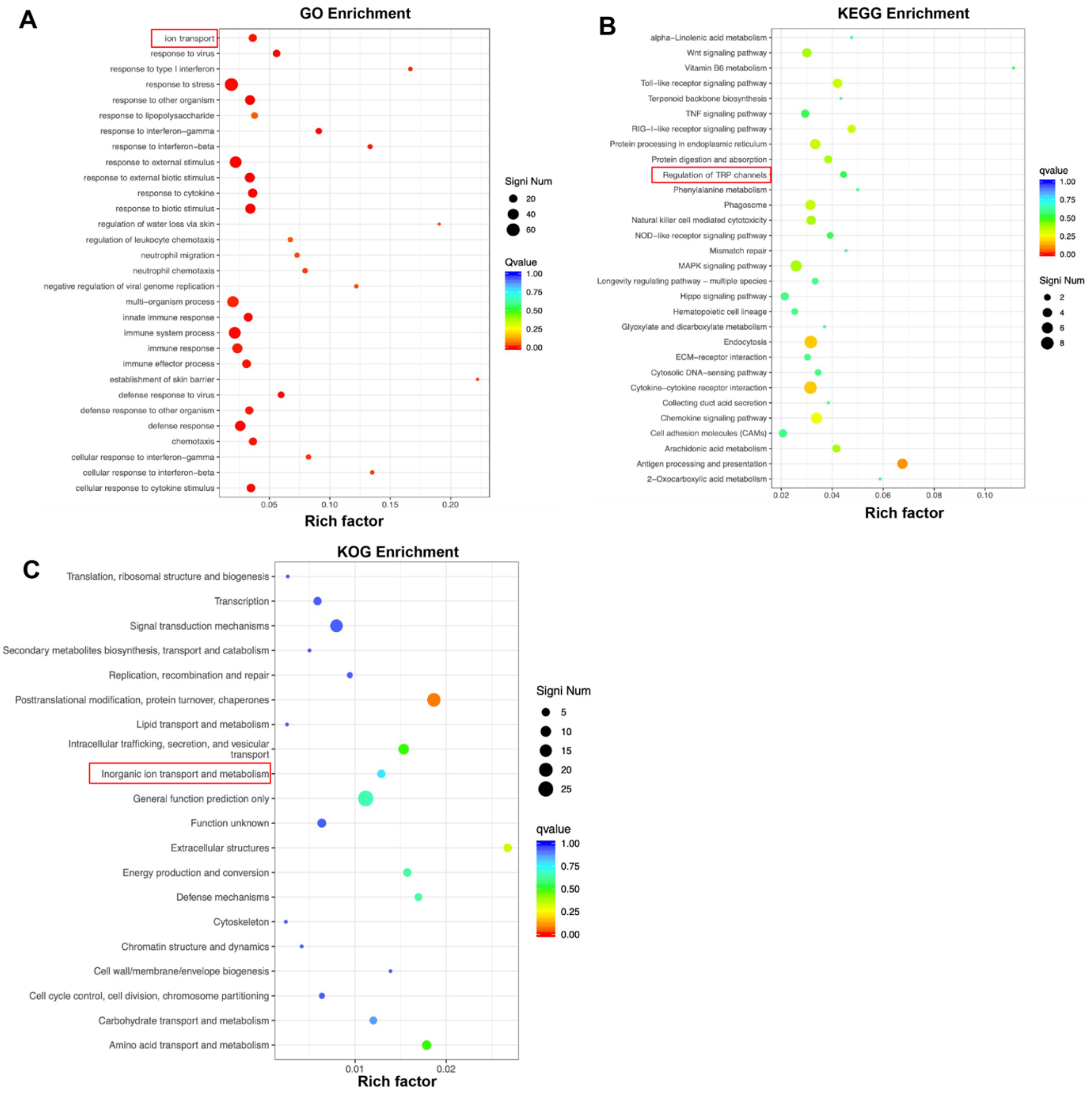

Figure S2. The results of RNA-seq.

(A) Results of the Gene ontology (GO) analysis.

(B) Results of the Kyoto Encyclopedia of Genes and Genomes (KEGG) analysis.

(C) Results of the euKaryotic Ortholog Groups (KOG) analysis.

Figure S3

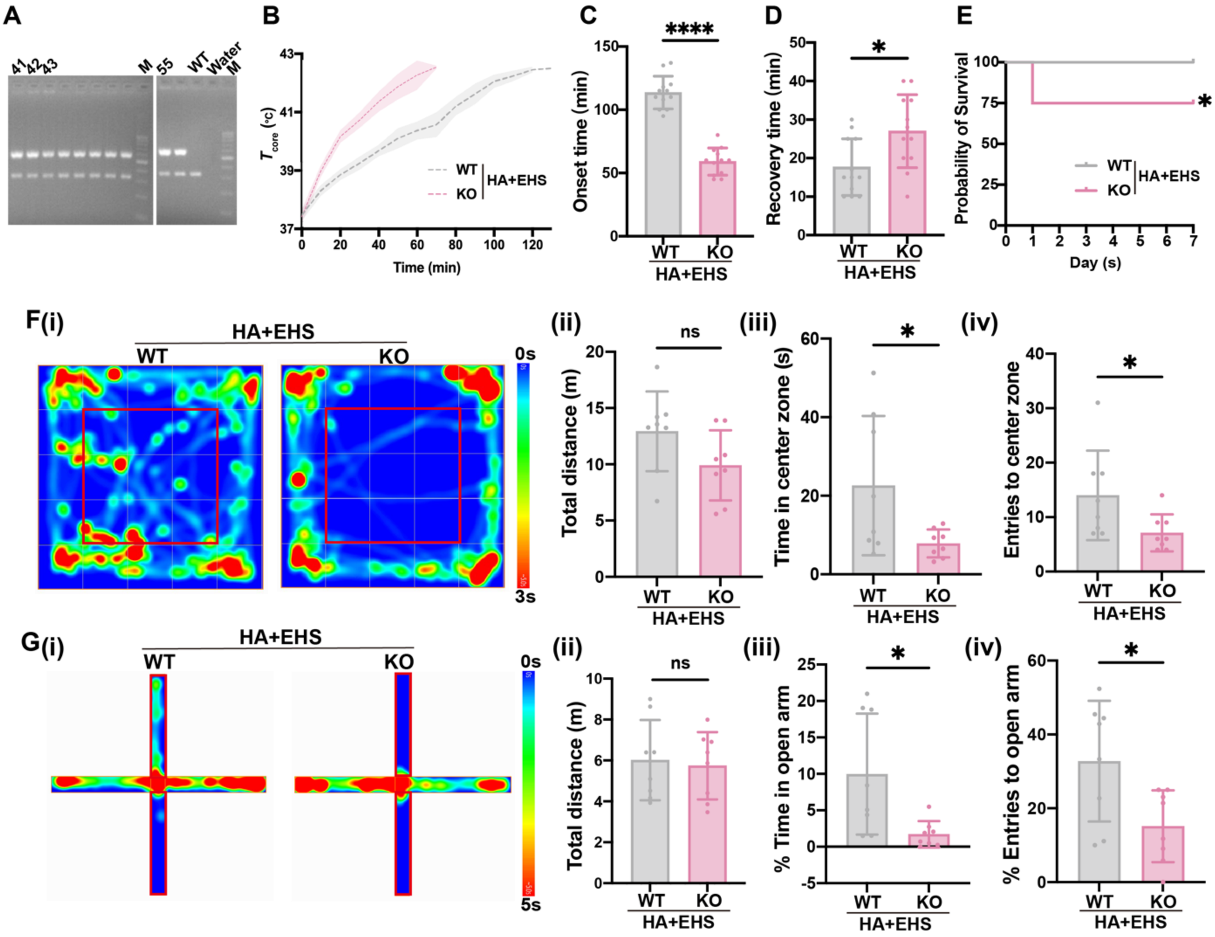

Figure S3. TRPV1 knockout reverses HA-triggered heat resistance.

(A) TRPV1-KO mice were identified positive by PCR screening.

(B) The changes of T_{core} during the modeling process.

(C-D) The onset time of heat-syncope (C) and recovery time post EHS (D) (n = 12).

(E) 7-day mortality rate in each group of mice (n = 12).

(F) Typical tracking chart of the OFT (i), statistics of total distance travelled (ii), time spent in the center zone (iii), and the number of entries to the center zone (iv) in the OFT at 48 h of recovery (n = 8).

(G) Typical tracking chart of the EPM (i), statistics of total distance travelled (ii), time spent in the open arm (iii), and the number of entries to the open arm (iv) in the EPM

at 48 h of recovery (n = 8).

All data are shown as mean ± SD and were analyzed by unpaired t-test. $*p < 0.05$, $**p < 0.01$, $***p < 0.001$, $****p < 0.0001$, ns, not significant.

Figure S4

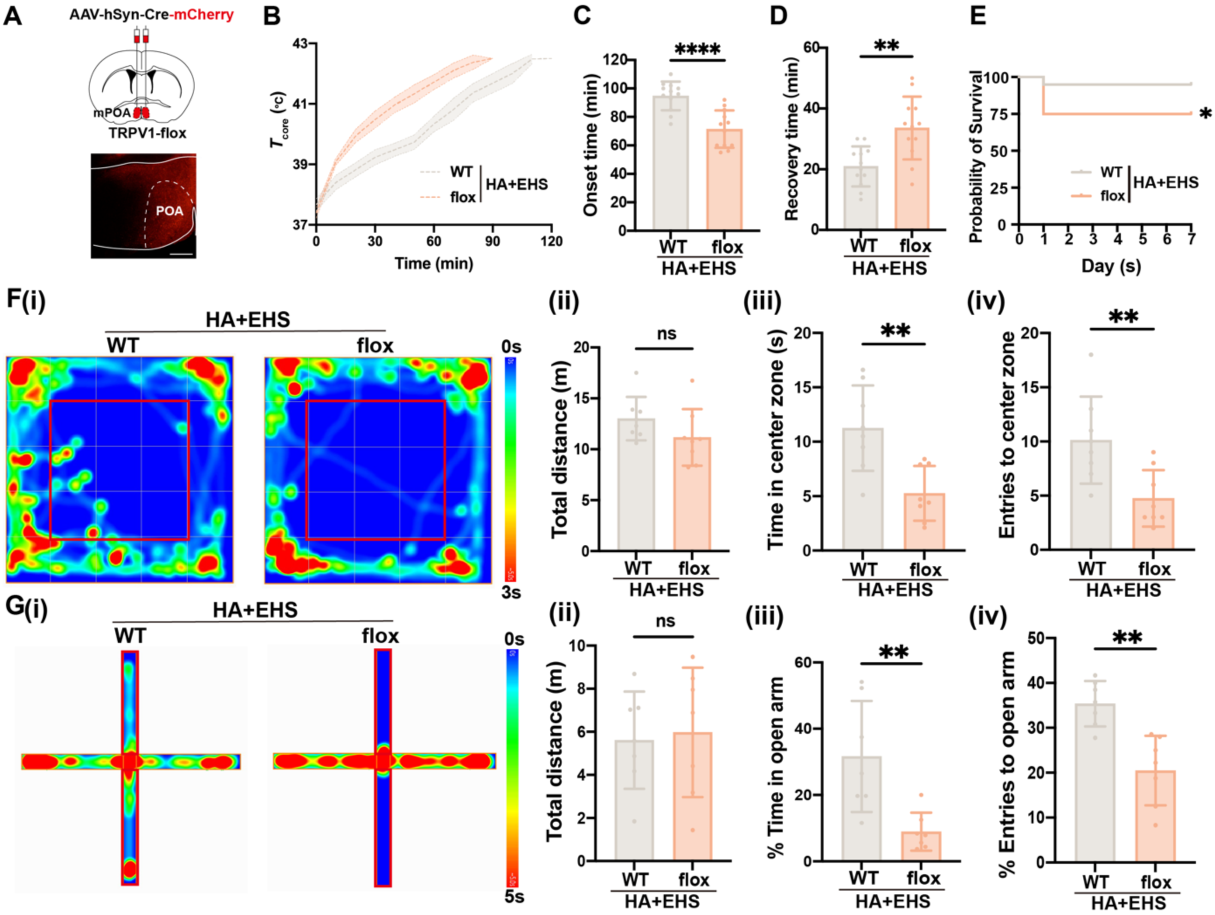

Figure S4. Selective TRPV1 knockout in the brain reverses HA-triggered heat resistance.

(A) Scheme and representative coronal images of the injection of AAV-hSyn-Cre-mCherry into mPOA. Scale bar, 100 μm.

(B) The changes of T_{core} during the modeling process.

(C-D) The onset time of heat-syncope (C) and recovery time post EHS (D) (n = 12).

(E) 7-day mortality rate in each group of mice (n = 20).

(F) Typical tracking chart of the OFT (i), statistics of total distance travelled (ii), time spent in the center zone (iii), and the number of entries to the center zone (iv) in the OFT at 48 h of recovery (n = 8).

(G) Typical tracking chart of the EPM (i), statistics of total distance travelled (ii), time spent in the open arm (iii), and the number of entries to the open arm (iv) in the EPM at 48 h of recovery (n = 8).

All data are shown as mean ± SD and were analyzed by unpaired t-test. *$p < 0.05$, **$p < 0.01$, ***$p < 0.001$, ****$p < 0.0001$, ns, not significant.

Figure S5

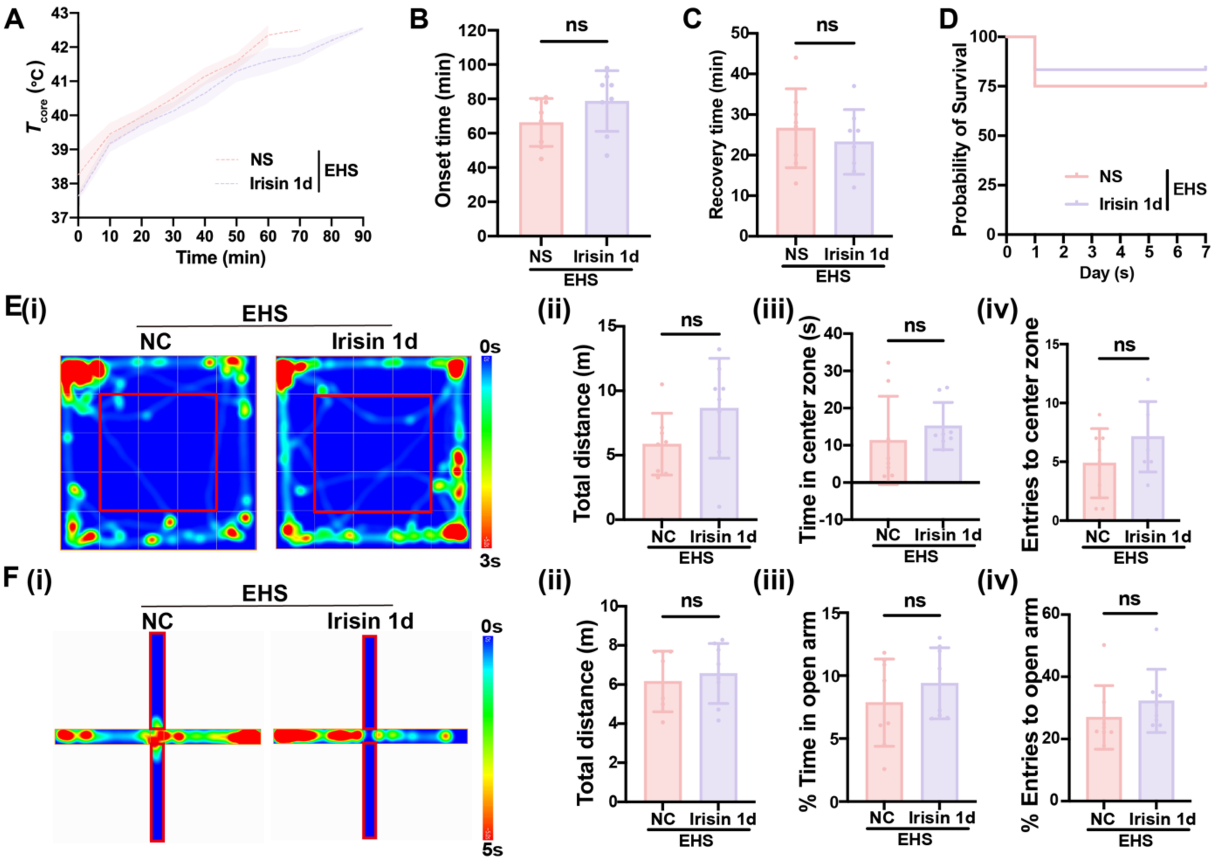

Figure S5. A single injection of irisin did not show significant protective effects.

(A) The changes of T_{core} during the modeling process.

(B-C) Statistics of the onset time of heat-syncope (B) and recovery time post EHS (C) (n = 8).

(D) Statistics of the 7-day mortality rate in each group of mice (n = 12).

(E) Typical tracking chart of the OFT (i), statistics of total distance travelled (ii), time spent in the center zone (iii), and the number of entries to the center zone (iv) in the OFT at 48 h of recovery (n = 8).

(F) Typical tracking chart of the EPM (i), statistics of total distance travelled (ii), time spent in the open arm (iii), and the number of entries to the open arm (iv) in the EPM at 48 h of recovery (n = 8).

All data are shown as mean ± SD and were analyzed by unpaired t-test. *$p < 0.05$, **$p < 0.01$, ***$p < 0.001$, ****$p < 0.0001$, ns, not significant.

Figure S6

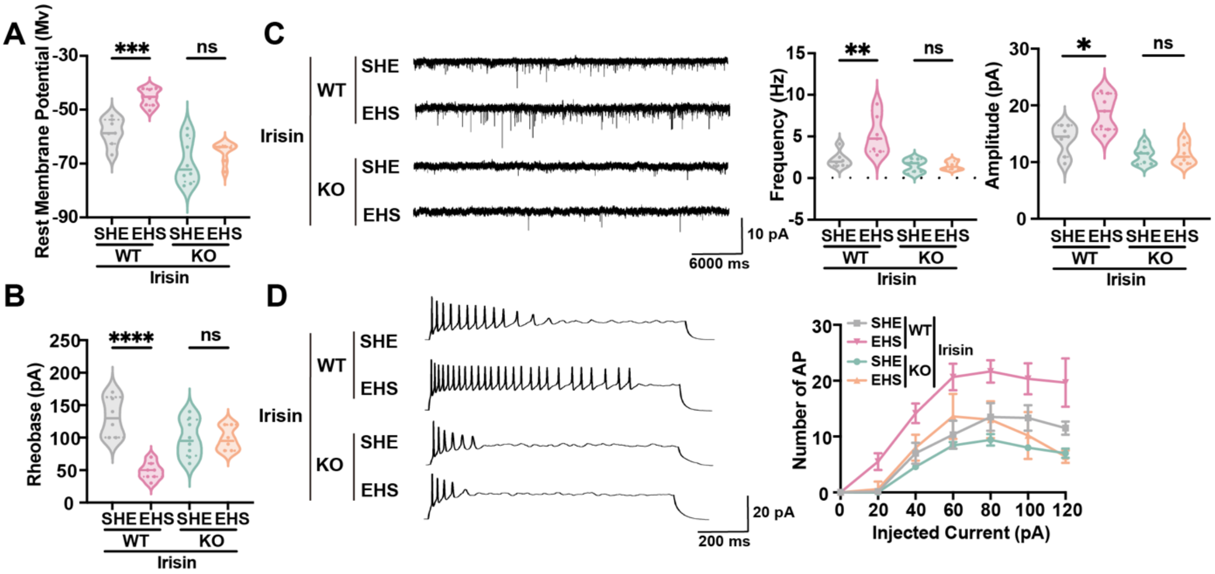

Figure S6. TRPV1 knockout inhibited EHS-mediated high excitability in neurons.

(A-B) Analysis of rest membrane potential and rheobase of $mPOA^{TRPV1}$ neuron in the SHE+WT+Irisin group (n = 7), the EHS+WT+Irisin group (n = 8), the SHE+KO+Irisin group (n = 8) and the EHS+KO+Irisin group (n = 7).

(C) Schematic illustration and the analysis of EPSP in the SHE+WT+Irisin group (n = 7), the EHS+WT+Irisin group (n = 8), the SHE+KO+Irisin group (n = 8) and the EHS+KO+Irisin group (n = 7).

(D) Schematic illustration and the analysis of AP in the SHE+WT+Irisin group (n = 5), the EHS+WT+Irisin group (n = 6), the SHE+KO+Irisin group (n = 5) and the EHS+KO+Irisin group (n = 6).

All data are shown as mean ± SD and were analyzed by one-way ANOVA, or two-way ANOVA. $^{*}p < 0.05$, $^{**}p < 0.01$, $^{***}p < 0.001$, $^{****}p < 0.0001$, ns, not significant.
